# Cardiac lymphatics retain LYVE-1-dependent macrophages during neonatal mouse heart regeneration

**DOI:** 10.1038/s44161-025-00711-4

**Published:** 2025-09-17

**Authors:** Benjamin G. Chapman, Konstantinos Klaourakis, Carla de Villiers, Mala Gunadasa-Rohling, Maria-Alexa Cosma, Susanna T. E. Cooper, Kshitij Mohan, Michael Weinberger, Carolyn A. Carr, David R. Greaves, David G. Jackson, Daniela Pezzolla, Robin P. Choudhury, Joaquim M. Vieira, Paul R. Riley

**Affiliations:** 1 Department of Physiology, Anatomy and Genetics, University of Oxford, Oxford, UK; 2 Institute of Developmental & Regenerative Medicine (IDRM), University of Oxford, Oxford, UK; 3 Division of Cardiovascular Medicine, Radcliffe Department of Medicine, University of Oxford, Oxford, UK; 4 Sir William Dunn School of Pathology, University of Oxford, Oxford, UK; 5 MRC Translational Immune Discovery Unit, MRC Weatherall Institute of Molecular Medicine, University of Oxford, Oxford, UK; 6 School of Cardiovascular & Metabolic Medicine & Sciences, King’s College London, London, UK

**Keywords:** Lymphangiogenesis, Cardiac regeneration

## Abstract

In adult mice, myocardial infarction (MI) activates the cardiac lymphatics, which undergo sprouting angiogenesis (lymphangiogenesis), drain interstitial fluid and traffic macrophages to mediastinal lymph nodes (MLNs). This prevents edema and reduces inflammatory/fibrotic immune cell content to improve cardiac function. Here we investigated the role of cardiac lymphatics and macrophage clearance across the neonatal mouse regenerative window. The response to injury revealed limited lymphangiogenesis and clearance of macrophages from postnatal day 1 compared to postnatal day 7 infarcted hearts. This coincides with the maturation of lymphatic endothelial cell junctions from impermeable to permeable and with altered signaling between lymphatic endothelial cells and macrophages. Mice lacking the lymphatic endothelial receptor-1 (LYVE-1), where macrophage lymphatic trafficking is impaired in adults, experienced worse long-term outcomes after MI induced at postnatal day 1, suggesting an alternative role for LYVE-1 in macrophages. Macrophage-specific deletion of *Lyve1* during neonatal heart injury impaired heart regeneration. This study demonstrates that immature cardiac lymphatics are impermeable to clearance in early neonates, ensuring retention of pro-regenerative LYVE-1-dependent macrophages.

## Abstract (web summary)

Chapman, Klaourakis and colleagues reveal that a lymphatic vasculature with poor clearance capacity in perinatal, regeneration-competent mouse hearts is required to retain pro-reparative macrophages and allow cardiac regeneration.

## Main

During adult homeostasis, the cardiac lymphatics function to modulate tissue fluid and immune surveillance, analogous to the systemic lymphatics that pervade the body. After injury, such as myocardial infarction (MI), they undergo lymphangiogenesis by growing and expanding into the infarcted area^1–4^. The epicardium and pro-inflammatory macrophages secrete vascular endothelial growth factor C (VEGFC), which drives lymphangiogenesis and extensive remodeling of the cardiac lymphatic network^2,3^. MI triggers an immune response, whereby infiltrating phagocytic cells, such as neutrophils and macrophages, remove dead tissue and debris, assisting with the subsequent remodeling and repair of the infarcted heart. High numbers and persistence of immune cells, however, contribute to further fibrosis and pathological remodeling and, ultimately, progression to heart failure (reviewed in ref. ^5^). The endogenous response to MI of increased lymphatic growth attempts to reduce tissue edema and immune cell load, targeting clearance of neutrophils, macrophages, dendritic cells and T cells, which is necessary for effective tissue repair^3,4^. However, the response is insufficient to clear the elevated fluid and excessive immune cell infiltrate, which cumulatively results in chronic inflammation, fibrosis and impaired heart function (reviewed in ref. ^6^). This has prompted attempts to increase lymphangiogenesis and lymphatic function in the injured heart. Augmentation of the lymphangiogenic response with administration of recombinant VEGFC-C156S, which specifically interacts with VEGFR3, improves cardiac function after MI in animal models, as assessed by echocardiography and cine magnetic resonance imaging (MRI)^2,3^. Intraperitoneal injection of recombinant VEGFC-C156S after MI in mice increased macrophage clearance via a LYVE-1-dependent mechanism^3^. LYVE-1 is highly expressed at the surface of initial lymphatics and interacts specifically with the ubiquitous glycosaminoglycan polymer hyaluronan that coats the surface of phagocytic immune cells, where it facilitates vessel entry^7–10^. Engagement of LYVE-1 with the long chains of hyaluronan involves an unusual sliding interaction that mediates the docking and transmigration of dendritic cells and macrophages to dermal lymphatic vessels^8,9,11,12^ and has a similar role in the injured adult mouse heart^3^.

In contrast to adult mammals, which are incapable of functional recovery after heart injury, the neonatal mammalian heart has an evolutionarily conserved regenerative capacity (reviewed in ref. ^13^). In mice, the heart fully regenerates after MI at postnatal day 1 (P1), whereas the same injury at postnatal day 7 (P7) leads to fibrotic scarring^14,15^. Anecdotal evidence from clinical case reports has described analogous cardiac regeneration in human infants after MI in utero caused by congenital heart disease^16,17^. In neonatal mice, the macrophages found in the intact heart at early postnatal stages are primarily tissue-resident CCR2^−^ macrophages that originate from embryonic sources and are maintained through local proliferation^18–20^. By contrast, circulating CCR2^+^ monocytes and monocyte-derived CCR2^+^ macrophages prevalent in adulthood contribute minimally to the cardiac monocyte–macrophage population at these stages^18,20^. In response to cardiac injury in neonates, tissue-resident CCR2^−^ macrophages expand in number, without additional infiltration of CCR2^+^ monocytes^18^. General depletion of macrophages following clodronate liposome treatment after MI at P1 inhibited cardiac regeneration, resulting in fibrotic scar formation with significantly depressed cardiac function^21^. This lack of regeneration was attributed to impaired coronary angiogenesis^21^, which is consistent with the growing evidence supporting direct and indirect macrophage contributions to blood vessel growth and vascular plexus formation^22^.

The essential function of tissue-resident macrophages in heart regeneration in neonatal mice^21^ together with the immunomodulatory role of lymphatic vessels in the adult mouse heart^3^ prompted our present investigation into how the cardiac lymphatics respond in the regenerative setting and to what extent the cardiac lymphatics interact with macrophages in injured neonatal mouse hearts across the P1–P7 window. The stage-dependent response of lymphatics and their trafficking of immune cells, specifically macrophages, during neonatal heart regeneration and the transition to fibrotic repair have not been studied to date. In the present study, we quantified the expansion of the lymphatic vascular network after birth to reveal growth and sprouting until postnatal day 16 (P16). We further investigated the lymphangiogenic response and macrophage trafficking efficiency of neonatal cardiac lymphatics after MI, using combined three-dimensional light-sheet and confocal imaging alongside adoptive transfer of splenic *hCD68–eGFP*-labeled monocytes. The injury response revealed a lack of lymphangiogenesis and less efficient clearance of GFP-labeled cells to MLNs 7 days post-injury (dpi) in P1 compared to P7 hearts. This was consistent with the need to retain pro-regenerative macrophages at P1 relative to invoking clearance of pro-fibrotic macrophages at P7, as recapitulates the adult injury and lymphatic response. The impaired clearance may be explained, in part, by the maturation status of lymphatic endothelial cell junctions and the developmental transition from ‘zippered’ (impermeable) to ‘buttoned’ (permeable) junctions during the first 2 weeks of life. To gain insight into the molecular underpinnings of lymphatic endothelium–macrophage interactions in P1 versus P7, we generated unbiased single-cell RNA sequencing (scRNA-seq) datasets from neonatal hearts collected at different timepoints after MI and observed altered lymphatic endothelial cell (LEC)–macrophage signaling. Notably, this included reduced expression of the lymphangiocrine factor reelin (RELN)^23^ in P7 versus P1 hearts, consistent with impaired regenerative potential. Finally, we explored the possible involvement of the lymphatic entry receptor LYVE-1 in early neonatal heart regeneration. Here, we anticipated that LYVE-1 would not play a role given that trafficking of pro-regenerative macrophages is redundant at early stages; however, we observed that global *Lyve1* deletion resulted in impaired regeneration, persistent scarring and loss of cardiac function, suggesting a function in a distinct lineage to that of the lymphatic endothelium. Conditional macrophage-specific targeting of *Lyve1* revealed that this dependence was due to a previously unappreciated role in tissue-resident macrophages, maintaining this population in situ to inhibit infiltrating pro-inflammatory/fibrotic monocytes and promote coronary angiogenesis to facilitate heart regeneration.

## Results

### Postnatal development of the cardiac lymphatics

**Extended Data Fig. 1:**
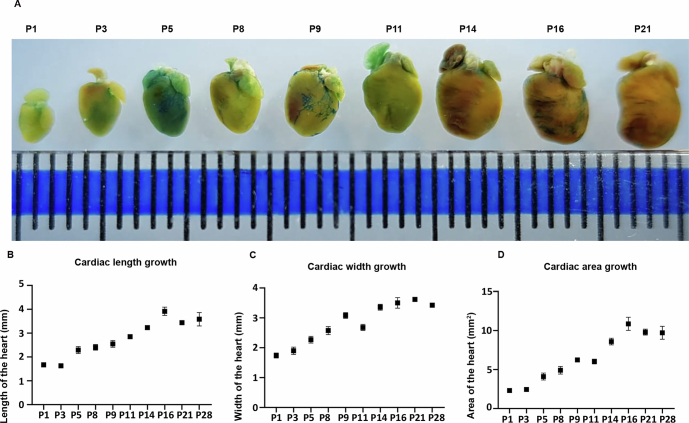
Heart growth during postnatal development. Hearts were harvested to quantify the length and width size across the ventral surface at different postnatal stages (**A**). The heart was found to grow from base to apex in proportion to increasing width and area (**B**-**D**). Four weeks after birth, the area of the heart had increased four-fold on the ventral side. Data are presented as mean ± SEM. n = 5 for P1; n = 3 for P3, P5, P9; n = 4 for P8, P11, P16, P21, P28; n = 10 for P14. Significant differences were calculated using 1-way ANOVA followed by Tukey’s multiple comparisons test. Scale bar: 1 mm. Source data

**Extended Data Fig. 2:**
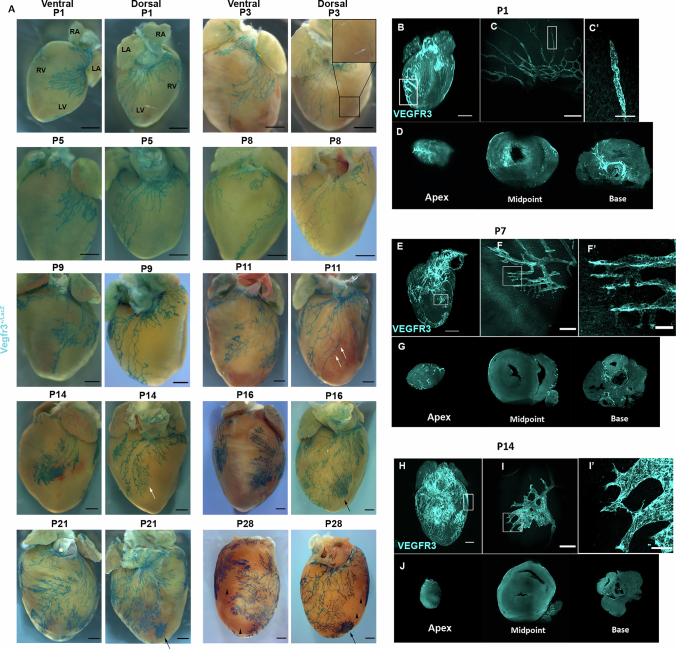
The cardiac lymphatic network undergoes expansion until late postnatal development. Whole-mount imaging of *Vegfr3*^*+/LacZ*^ hearts showed extensive growth and sprouting of the cardiac lymphatic vasculature on both the dorsal and ventral surfaces of the heart during postnatal stages (**A**). Isolated LECs were present on the dorsal side of the heart during early postnatal stages (grey arrows at P1, P3) and by P11 and P14 sprouts were detected arising from a large dorsal lymphatic vessel (white arrows). These sprouts produced a dense plexus of vessels near the apex at P16 (black arrow at P16 - P28). Areas depleted of lymphatics were present in stereotypical locations of the heart, such as the apex on the ventral side (black arrowheads at P28) and the right ventricle on the dorsal side (black arrowheads at P28). Whole mount hearts were cleared using iDISCO at P1, P7, and P14 and stained for VEGFR3 before imaging with Lightsheet microscopy (**B**-**J**). Initial lymphatics were identified morphologically (**C**, **C**’, **F**, **F**’, **I**, **I**’). Representative sections (**D**, **G**, **J**) from the 3-dimensional images (**B**, **E**, **H**) illustrate lymphatics are largely absent at depth. n = 3-5 for each time point in A. Scale bars: 0.5 mm for **A**, **B**, **E**, **H**; 200μm for **C**, **F**, **I**; 50μm for magnified views.

**Extended Data Fig. 3:**
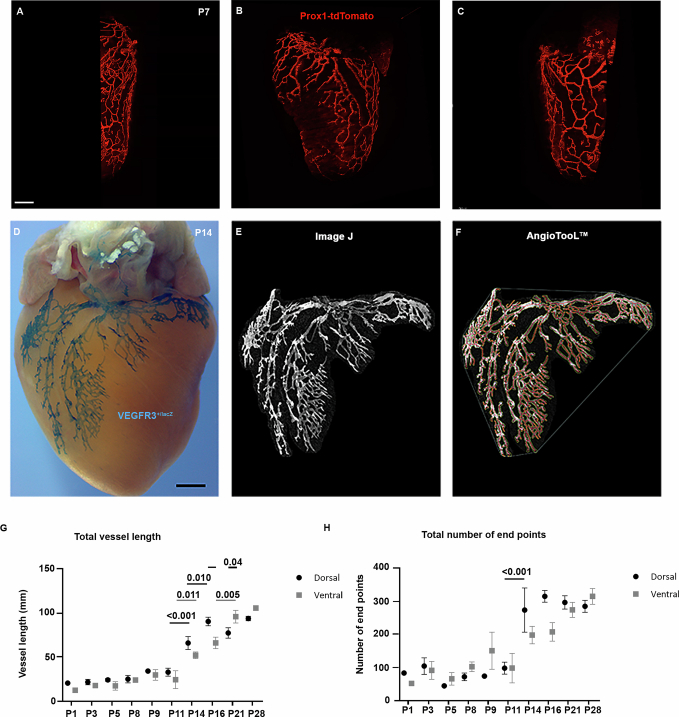
Imaging-based assessment and quantification of the post-natal cardiac lymphatic plexus. Light-sheet and wholemount confocal imaging of *Prox1-tdTomato* (**A**-**C**) and *Vegfr3*^*+/lacZ*^ (**D**) intact hearts, at post-natal days 7 and 14 respectively, enable visualisation of the cardiac lymphatic plexus which can be segmented using Image J (**E**) and quantified in terms of parameters such as vessel length, calibre, junctions, branch points etc using AngioTool^TM^ (**F**). The total vessel length and number of end points remained stable until P14 and then significantly increased on both sides of the heart via lymphangiogenesis (**G**). By P16 the lymphatics were fully formed on the dorsal side, while the ventral lymphatics were delayed, reaching full expansion by P21 (**H**). Data are presented as mean ± SEM. n = 5 for P1, P16, P21; n = 3 for P3, P5, P9; n = 4 for P8, P11, P28. Significant differences were calculated using 2-way ANOVA. Scale bars 0.5 mm, A and 1 mm, D. Source data

The heart grows substantially during postnatal development, from an average area of 2.5 mm^2^ at P1 to 10 mm^2^ at P16 (Extended Data Fig. 1). To visualize the lymphatic network, we used 5-bromo-4-chloro-3-indolyl-β-d-galactosidase (X-Gal) staining of the knock-in mouse line *Vegfr3*^*+/LacZ*^ (ref. ^24^), which revealed a concordant expansion of the network across postnatal stages P1–P28 (Extended Data Fig. 2a). At P1, lymphatics were located near the base of the heart on the ventral side, whereas, on the dorsal side, they extended toward the apex, proximal to the major coronary veins. During the first week of postnatal development (from P1 to P9), no sprouting of new lymphatic vessels was observed on either the ventral or the dorsal side of the heart, as quantified by three-dimensional rendering of confocal and light-sheet images and use of ImageJ and AngioTool software (Extended Data Fig. 3). During this period, cardiac lymphatic length increased with organ growth to establish full coverage from base to apex on the dorsal side and incomplete coverage on the ventral side of the heart. The reduced length and coverage of vessels on the ventral versus dorsal side is consistent with delayed lymphatic development during embryonic stages^1^. Also, a small number of isolated VEGFR3^+^ cells were noticeable on the dorsal side of the heart, which were not connected to the already established lymphatic vasculature at P1 and P3. These resembled the isolated LECs that have been described to contribute to the cardiac lymphatic network in zebrafish and mammals through a process termed lymphvasculogenesis^25^. From P11, the first sprouts of vessels were visible on both the sides of the heart. These sprouts continued expanding until they formed a complex network of vessels that appeared fully developed by P28. This pattern of lymphatic growth was consistent across samples, becoming established via a dense network of vessels on the dorsal side near the apex at P14 and fully shaped by P28. A further constant feature was the presence of areas that were depleted of lymphatics, such as the apex on the ventral side and the right ventricle on the dorsal side.

**Fig. 1:**
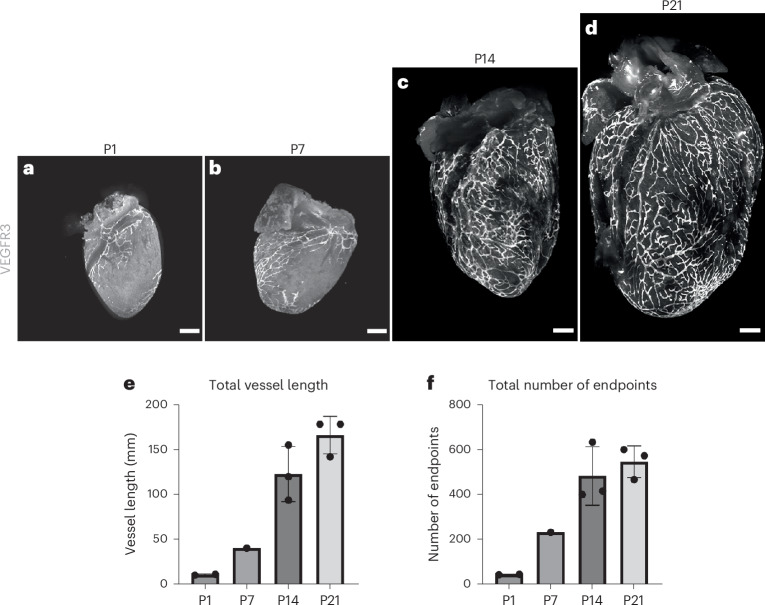
Growth of the lymphatic network from P1 to P21. Whole-mount antibody staining for VEGFR3 confirmed that cardiac lymphatics grow and sprout extensively during postnatal development. Although little growth or sprouting was observed in the first week of postnatal life (**a**,**b**), by P14 a dense network of lymphatic vessels had formed covering the entire dorsal side of the heart (**c**). This network continued growing in proportion to overall heart expansion (**d**). Quantification of cardiac lymphatics on the dorsal side confirmed that there is a significant increase in total vessel length and total number of endpoints during the second week of life (**e**,**f**). Data are presented as mean ± s.e.m.; *n* = 5 for each timepoint. Scale bar, 500 μm. Source data

Immunostaining for VEGFR3 in CD1 wild-type (WT) hearts was carried out to validate the results from the X-Gal staining of *Vegfr3*^*+/LacZ*^ mice at different postnatal stages (Fig. 1a–d). VEGFR3^+^ lymphatics fully covered the heart by P14, indicating that sprouting occurred at an earlier timepoint and with greater efficiency. Moreover, lymphatics on the dorsal side of CD1 hearts continued expanding concurrently with cardiac growth at least until P21. Further quantification of VEGFR3-immunostained lymphatics from the dorsal side hearts revealed an increase in the total vessel length from 15 mm at P1 to 175 mm at P21 (Fig. 1e), in line with continuous growth, and the total number of endpoints increased from 50 vessels at P1 to 500 vessels at P14, indicating increased complexity of the network (Fig. 1f). iDISCO optical clearing was used to assess lymphatic structure at depth in uninjured WT hearts at P1, P7 and P14 (Extended Data Fig. 2b–j). This analysis confirmed that lymphatics are present mostly in the outer cardiac surface throughout the regenerative period and supported the examination of lymphangiogenesis by whole-mount staining (Fig. 1a–d).

Comparing the expanding networks on the dorsal and ventral sides of the heart revealed different growth dynamics across the postnatal period (Extended Data Fig. 3g,h). The mean length and number of individual vessels increased marginally from P1 (dorsal: 21 mm and 83 vessels; ventral 13 mm and 52 vessels) to P11 (dorsal: 33 mm and 98 vessels; ventral 25 mm and 98 vessels), suggesting limited growth and sprouting. Consistent with the visualization of lymphatic sprouting after P11, there was a significant increase in total vessel length on the dorsal side from 33 mm at P11 to 66 mm at P14 (*P* < 0.001) and from 66 mm at P14 to 91 mm at P16 (*P* < 0.01). On the ventral side, vessel length increased from 25 mm at P11 to 52 mm at P14 (*P* < 0.011) and from 66 mm at P16 to 96 mm at P21 (*P* < 0.005). Also, the total number of endpoints increased significantly on the dorsal side, from 98 vessels at P11 to 273 vessels at P14 (*P* < 0.001). A trend toward smaller total vessel length on the ventral side was observed throughout early postnatal development, which was statistically significant at P16 with 91-mm vessel length on the dorsal compared to 66-mm vessel length on the ventral side (*P* = 0.009).

**Extended Data Fig. 4:**
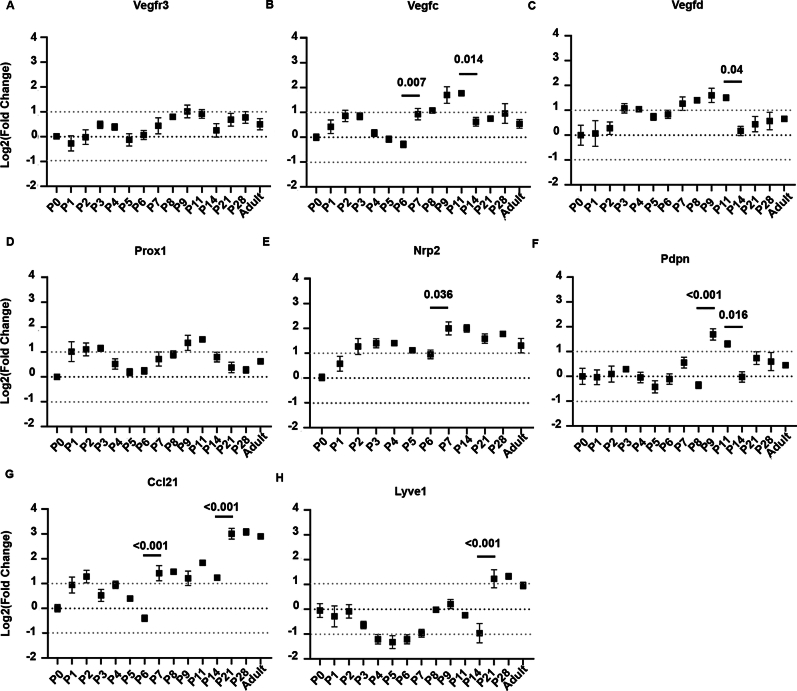
Expression of lymphatic markers across the post-natal period. Whole-heart samples across post-natal stages P0-P28 were analysed by qPCR for lymphatic markers. Expression levels of genes implicated in lymphangiogenesis, such as *Vegfr3* (**A**), *Vegfc* (**B**), *Vegfd* (**C**), *Prox1* (**D**) and *Nrp2* (**E**) revealed biphasic increases, with the first between P2 and P3 and a second around P9. *Pdpn* increased only between P9 and P11 (**F**). Genes implicated in lymphatic function, such as *Ccl21* (**G**) and *Lyve1* (**H**) displayed an expression pattern where levels fluctuated during the first week, before becoming increasing significantly at P21 and thereafter remained stable. Data are presented as mean ± SEM; n = 3 for each time point. Significant differences were calculated using 1-way ANOVA followed by Tukey’s multiple comparisons test. Source data

To uncover potential molecular changes in the lymphatics during postnatal development, quantitative polymerase chain reaction (qPCR) was conducted using primers specific for a panel of known lymphatic markers and whole-heart samples (Extended Data Fig. 4). The gene expression levels for the majority of lymphangiogenic markers revealed two peaks during postnatal development, one at P2–P3 and one at P7–P9 (Extended Data Fig. 4a–e). At P2–P3, the expression levels of *Prox1* and *Nrp2* and the ligands *Vegfc* and *Vegfd* doubled compared to P0. Subsequently, expression levels dropped for *Prox1*, *Vegfc* and *Vegfd* and remained stable for *Nrp2* until P6. Thereafter, expression levels of *Vegfc* (*P* = 0.007) and *Nrp2* (*P* = 0.036) increased significantly from P6 to P7, and *Vegfr3*, *Vegfd* and *Prox1* doubled at P8–P9 compared to P0. After P11, the expression levels of all genes decreased, except for *Nrp2*, which remained elevated. Specifically, expression of *Vegfc* (*P* = 0.014) and *Vegfd* (*P* = 0.04) declined significantly from P11 to P14, and expression of *Vegfr3* and *Prox1* returned to nearly P0 levels. Expression of *Pdpn* remained relatively stable during postnatal stages (Extended Data Fig. 4f), with a significant increase (*P* < 0.001) at P9 compared to P8 and a subsequent significant decrease (*P* = 0.016) to baseline levels at P14 compared to P11. The increased expression of lymphatic markers at P2–P3 correlates with the expansion of cardiac lymphatics observed with neonatal heart growth, whereas the increased expression levels at P8–P11 correlated with the lymphangiogenic sprouting observed after P11.

Analysis of genes involved in lymphatic function, rather than development, revealed a different pattern (Extended Data Fig. 4g,h). For example, the expression levels of *Ccl21* (Extended Data Fig. 4g), the gene coding for the LEC-secreted chemokine CCL21 (chemokine (C-C motif) ligand 21), responsible for recruitment and lymphatic endothelial transmigration of leukocytes into the lymph^8,26,27^, increased two-fold at P2 compared to P0 and returned to baseline levels by P6. At P7, there was a further significant increase (*P* < 0.001), which remained stable until P14, after which there was another significant increase (*P* < 0.001) at P21. From P21 until adulthood, the expression levels of *Ccl21* remained stable, with an eight-fold increase compared to the baseline. Similarly, expression of *Lyve1* increased significantly (*P* < 0.001) at P21 compared to P14 and remained stable into adulthood, with a two-fold increase compared to P0 (Extended Data Fig. 4h). *Lyve1* was the only gene to decrease two-fold in expression levels between P3 and P7.

Taken together, these results reveal the expansion of the lymphatic network and concomitant molecular changes during postnatal development and highlight different spatiotemporal behavior of lymphatic vessels on dorsal versus ventral sides of the heart coincident with functional maturation at later stages (P28).

### The cardiac lymphatics respond differently to MI during regenerative (P1) versus fibrotic (P7) wound healing

**Fig. 2:**
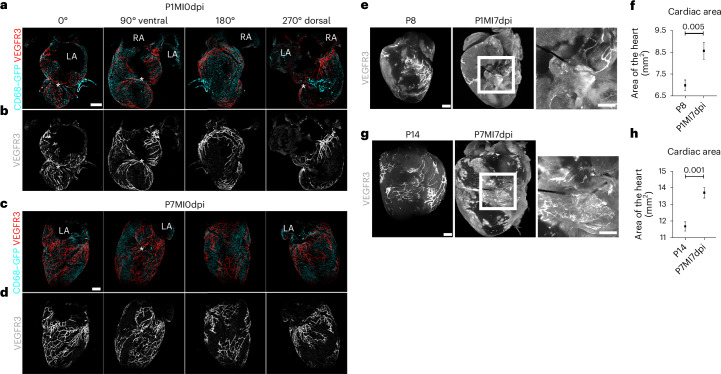
P1 versus P7 cardiac lymphatic responses immediately following MI and 7 days after MI compared to intact P8 and P14 hearts. Injured hearts were harvested at P1 and P7 immediately after MI surgery to visualize the initial response of the lymphatic vessels and macrophages (**a**,**c**). Macrophages were evenly distributed across all areas of the heart, suggesting that the immune response to injury had not yet fully initiated (**a**,**c**). Lymphatics extended from the base to apex in both P1 (**b**) and P7 (**d**) hearts. During ligation, the suture captured lymphatics located in the area of injury (asterisk in second panel of **b** and **d**). Whole-mount immunostaining of C57BL/6 hearts for VEGFR3, combined with light-sheet imaging (**e**,**g**). Intact P8 hearts compared to 7 days after MI at P1 revealed a limited lymphangiogenic response after injury (**e**). By contrast, comparing P14 intact with P7 hearts 7 days after MI revealed expanded VEGFR3^+^ lymphatic vessels covering the injury site (**g**). There was a significant increase in the heart size after MI at both P1 and P7 compared to the respective control stage (**f**,**h**). Asterisk indicates suture site. LA, left atria; RA, right atria. Data are presented as mean ± s.e.m. *n* = 2 for each timepoint (**a**–**d**); *n* = 12 for P8, *n* = 4 for P1MI7dpi in **f**; *n* = 7 for P14, *n* = 4 for P7MI7dpi in **h**. Significant differences were calculated using an unpaired, two-tailed Student’s *t*-test. Scale bar, 0.5 mm. Source data

To examine the response of neonatal cardiac lymphatics after MI, the left anterior descending (LAD) coronary artery was surgically ligated in *hCd68–eGFP* macrophage reporter at either P1 or P7 stages. Initially, hearts were harvested within 30–60 minutes after LAD ligation, termed 0 dpi, and immunostained for VEGFR3 to visualize the lymphatic vasculature in conjunction with hCD68–eGFP^+^ macrophages. The lymphatic vessels expanded from the base of the heart to the site of injury at 0 dpi in both P1 and P7 stages (Fig. 2a–d). The developing lymphatic network had more vessels and was morphologically more complex at P7 than at P1 (compare Fig. 2b to Fig. 2d). At 0 dpi, the macrophage response had not yet initiated, as there was no obvious change in hCD68–eGFP^+^ macrophage representation (compare Fig. 2a and Fig. 2c).

In adult mice, cardiac lymphatics respond to the site of injury by 7 dpi^1–3^. Thus, infarcted P1 and P7 hearts were sampled 7 days after surgery and compared to equivalent intact (non-infarcted) stages (P8 and P14; Fig. 2e–h). Whole-mount light-sheet imaging of hearts stained for VEGFR3 revealed limited lymphangiogenic response at the site of injury after P1 MI compared to intact age-matched control P8 hearts (Fig. 2e,f). Higher magnification revealed a limited presence of lymphatics proximal to the suture site in infarcted P1 hearts; a large vessel expanding from below the left atria to the injury site was consistently present, but this was also evident in P8 intact hearts, suggesting that this did not form in response to injury (Fig. 2e). By contrast, there was a clear lymphatic response in P7 MI hearts at 7 dpi, and higher magnification revealed an expansion of lymphatics surrounding the suture area (Fig. 2g). The remainder of the lymphatic network on both dorsal and ventral sides of the heart appeared underdeveloped in P7 infarcted hearts at 7 days compared to P14 intact controls (Fig. 2g). These results suggest a localized lymphangiogenic response at the site of injury, whereas the normal developmental program is delayed or compromised in remote areas, reflecting the global impact of myocardial ischemia. Notably, the size of hearts was significantly larger 7 days after MI in both P1 and P7 mice compared to the intact age-matched control P8 and P14 mice (compare Fig. 2f and Fig. 2h). This is likely due to myocardial hypertrophy and/or increased edema resulting from the cardiac injury, as previously reported in the adult mouse heart^2^.

**Fig. 3:**
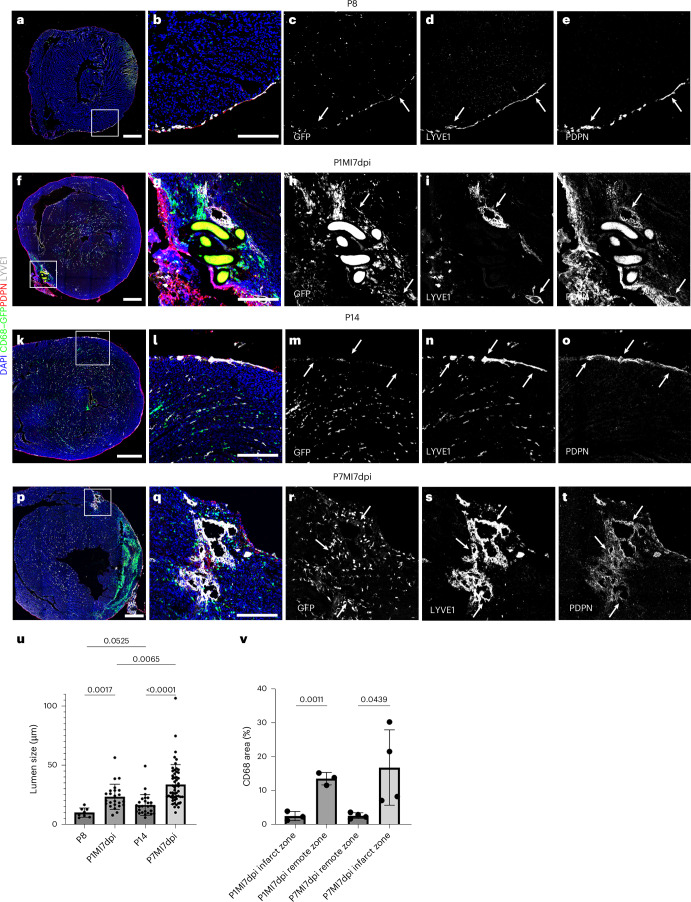
Lymphatic vessel expansion and macrophage accumulation at the site of injury. Serial sections and immunostaining for PDPN and LYVE-1 in intact P8 (**a**–**e**) versus injured hearts at P1 (day 7 after MI; **f**–**j**) and in intact P14 (**k**–**o**) versus injured hearts at P7 (day 7 after MI; **p**–**t**) confirmed the limited lymphangiogenic response relative to intact P8 and P14 controls (compare **a**–**e** to **f**–**j** and **k**–**o** to **p**–**t**) after MI at P1 compared to P7 (compare **g**–**j** to **q**–**t**). There was an increased number of lymphatic vessels with dilated lumen in P7 MI samples compared to the P14 intact controls (compare **b** to **g** and **l** to **q**). hCD68–eGFP^+^ macrophages were enriched at the site of injury after MI at P1 and P7 (**h** and **r**). Suture is visible through autofluorescence in **f**–**h** and **j**. Quantification of cardiac lymphatic lumen (**u**). Quantification of macrophage density (**v**). **b**–**e**, magnified view of **a** box; **g**–**j**, magnified view of **f** box; **l**–**o**, magnified view of **k** box; **q**–**t**, magnified view of **p** box. Data are presented as mean ± s.e.m. *n* = 2 hearts for P8, P1MI7dpi and P7MI7dpi and *n* = 3 hearts for P14 in **u**. The mean lumen size per timepoint was calculated from pooled results across hearts as technical replicates. *n* = 8, 27, 24 and 53 for P8, P1MI7dpi, P14 and P7MI7dpi, respectively, and *n* = 3 for P1MI7dpi in **v** and *n* = 4 for P7MI7dpi in **v**. Significant differences were calculated using one-way ANOVA followed by Tukey’s multiple comparisons test. Scale bar, 0.5 mm for **a**, **f**, **k** and **p**; 0.2 mm for magnified views. Source data

To further visualize the response to MI at P1 versus P7 stages and to document the macrophage response, *hCD68–eGFP* mice were subjected to MI surgery, and transverse serial sections of injured versus intact hearts were stained for lymphatic markers PDPN and LYVE-1 and imaged using confocal microscopy (Fig. 3). In both P8 and P14 intact hearts, lymphatics were found widely distributed throughout the subepicardial space of the heart, and GFP^+^ macrophages were not co-localized with the vessels (compare Fig. 3a–e and Fig. 3k–o). After injury, a moderate PDPN^+^ and LYVE-1^+^ lymphangiogenic response was seen at P1 (Fig. 3f–j), which was more substantial after injury at P7 (Fig. 3p–t), consistent with the different levels of response observed in whole-mount imaged hearts (Fig. 2). Lymphatic vessels were found near the suture site after MI at both P1 and P7; however, they were denser and more expanded in terms of lumen size in the infarcted P7 hearts (Fig. 3u; also compare Fig. 3f–j to Fig. 3p–t). MI at both P1 and P7 resulted in an increased concentration of GFP^+^ macrophages at 7 days, which were extensively localized proximal to the site of injury as compared to P8 or P14 controls (compare Fig. 3h to Fig. 3c and Fig. 3p–t to Fig. 3k–o; Fig. 3v).

Collectively, these results revealed modest lymphangiogenesis after MI at P1, whereas, at P7, the expansion of the lymphatic vessels proximal to the infarct was significant, mimicking the adult heart response^1,3^. Injury at both stages resulted in an accumulation of CD68^+^ macrophages, co-localized with the lymphatic vessels at the injury site.

### The cardiac lymphatics at P1 do not function to clear macrophages after injury

**Fig. 4:**
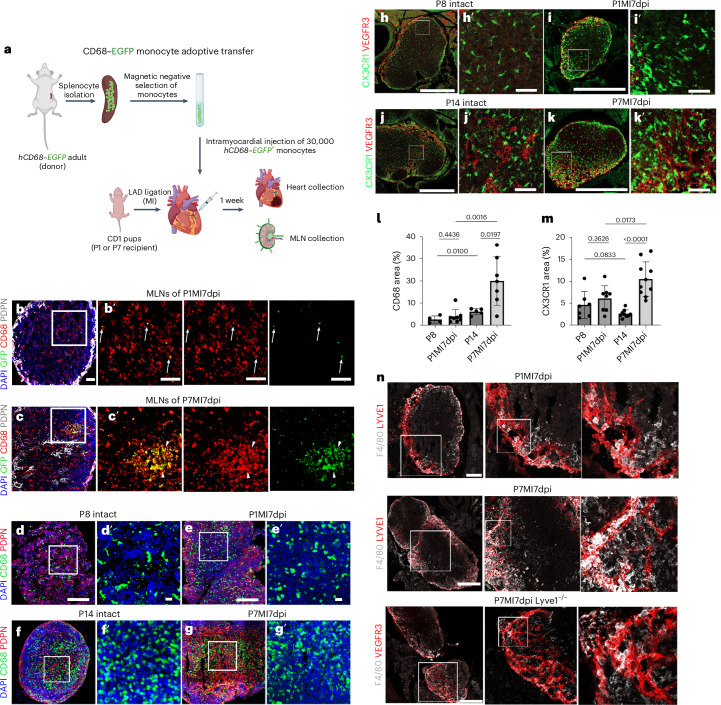
Adoptive transfer of splenic hCD68–GFP^+^ monocytes and imaging of CX3CR1^+^ tissue-resident macrophages reveals different levels of clearance to MLNs after MI at P1 versus P7. Schematic of the adoptive cell transfer approach using adult *hCD68–eGFP* transgenic mice as splenic GFP^+^ monocyte donors, for intramyocardial delivery into recipient neonatal CD1 mice at the time of MI surgery to assess immune cell trafficking (**a**). Immunostaining for CD68 and endogenous GFP fluorescence in tissue sections derived from MLNs of P1 and P7 mice that underwent MI, determining the presence of cleared CD68^+^GFP^+^ macrophages (**b′** white arrows and **c′** white arrowheads). CD68^+^GFP^+^ macrophages were substantially reduced in MLNs after MI at P1 compared to after MI at P7 (compare **b** and **c**). Visualization of endogenous GFP^+^ macrophages in MLNs from *hCD68–eGFP* mice confirmed minimal clearance at P1 after MI, which appeared increased at P7, compared to the respective intact controls that contained resident MLN GFP^+^ macrophages (compare **d** and **e** and compare **f** and **g**). Similar visualization in *CX3CR1–eGFP* mice also confirmed minimal clearance at P1 after MI, which increased at P7 (compare **h** and **i** and compare **k** and **j**). Quantification of macrophage numbers in the MLNs validated these observations and indicated that the difference in clearance at P1 versus P7 was significant (**l**,**m**). F4/80^+^ macrophages visualized within afferent lymphatic lumens of MLNs after MI at P7 but not at P1 and evidence of macrophage drainage disruption after P7 MI in the *Lyve1*^−*/*−^ mutant setting (**n**). **b′**–**k′** indicate magnified view of panel boxes. Data are presented as mean ± s.e.m. In **l**, *n* = 4 for P8, *n* = 8 for P1MI7dpi, *n* = 5 for P14 and *n* = 7 for P7MI7dpi. In **m**, *n* = 7 for P8, *n* = 8 for P1MI7dpi, *n* = 9 for P14 and *n* = 10 for P7MI7dpi. Magnification boxes are illustrative. Quantification was conducted across the entire MLN area within 10-μm sections. Significant differences were calculated using one-way ANOVA followed by Tukey’s multiple comparisons test. Scale bars, 50 μm for **b** and **c**; 0.5 mm for **d**–**k**; 20 μm for **d′**–**k′**; and 250 μm for **n**. Source data

**Extended Data Fig. 5:**
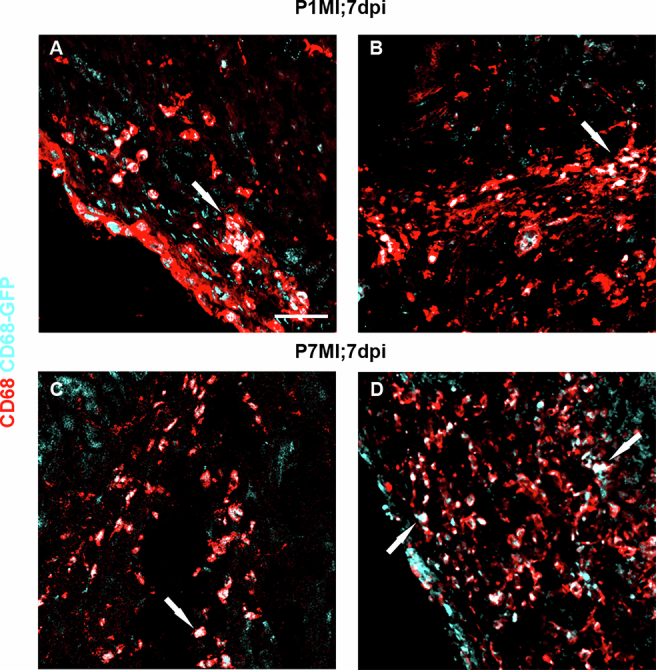
Successful engraftment of CD68 + GFP+ monocytes in the heart after adoptive cell transfer. GFP and CD68 immunostaining of cardiac tissue sections revealed appropriate engraftment of CD68^+^GFP^+^ monocytes within the injury area of the heart 7 days after adoptive transfer concurrent with MI at P1 and P7 (white arrows) (A-D). Two representative hearts for each condition shown. n = 4 hearts analysed across each stage. Scale bar: 50 μm.

To assess whether the neonatal cardiac lymphatics function to clear macrophages from the injured heart to draining lymph nodes, we carried out adoptive cell transfer of *hCD68–eGFP* monocytes into injured WT donors. Splenic *hCD68–eGFP* monocytes were isolated from adult mice, to ensure that they were competent to be trafficked, and approximately 3 × 10^4^ cells were injected into the myocardium of CD1 P1 and P7 recipient mice at the time of MI surgery (Fig. 4a). The hearts and MLNs were harvested at 7 dpi and imaged to identify both engrafted and cleared GFP^+^ macrophages (Fig. 4a). GFP^+^ macrophages were present in all hearts and were located predominately at the site of injury in both P1 and P7 mice, confirming successful adoptive transfer and engraftment (Extended Data Fig. 5a–d).

PDPN staining was used to confirm the identification of the MLNs in early postnatal mice and was localized to both the periphery and central areas of the isolated lymph nodes (Fig. 4b,c). MLNs were also stained with an antibody against the pan-monocyte/macrophage marker CD68, which revealed that only a small number of GFP^+^ macrophages were identified in the MLNs of mice that underwent MI at P1 (Fig. 4b′), whereas large clusters of GFP^+^ macrophages were evident in the MLNs of mice that underwent MI at P7 (Fig. 4c′).

To further validate the adoptive transfer data, and to extend the analysis more specifically to tissue-resident macrophages, MLNs were isolated from *hCD68–eGFP* and *CX3CR1–eGFP* injured and control mice. *CX3CR1–eGFP* was previously shown to mark tissue-resident macrophages (reviewed in ref. ^28^). The MLNs were sectioned, and images were acquired for quantification of the CD68^+^ or CX3CR1^+^ macrophage numbers in relation to the lymph node size (Fig. 4d–k). The presence of both CD68^+^ and CX3CR1^+^ macrophages in MLNs 7 days after MI at P1 was not significantly different compared to intact P8 hearts (compare Fig. 4d′ to Fig. 4e′ and Fig. 4h′ to Fig. 4i′; Fig. 4l,m), whereas CD68^+^ and CX3CR1^+^ macrophage numbers were significantly elevated after MI at P7 (20.1% and 10.6%) compared to P14 intact hearts (6.4% and 2.8%) (*P* < 0.05) (compare Fig. 4f′ to Fig. 4g′ and Fig. 4j′ to Fig. 4k′; Fig. 4l,m). Staining for F4/80 facilitated delineation of macrophages from dendritic cells and confirmed the increased presence of macrophages within the afferent lymphatics, draining to the MLNs, at 7 days after MI at P7 but not at P1 (Fig. 4n). Furthermore, MLNs were also examined in *Lyve1* knockout (KO) mice 7 days after MI at P7, when macrophages are observed to accumulate in the WT setting (Fig. 4n), revealing disrupted lymphatic trafficking and an absence of macrophages within the afferent lymphatic lumen compared to WT controls.

**Extended Data Fig. 6:**
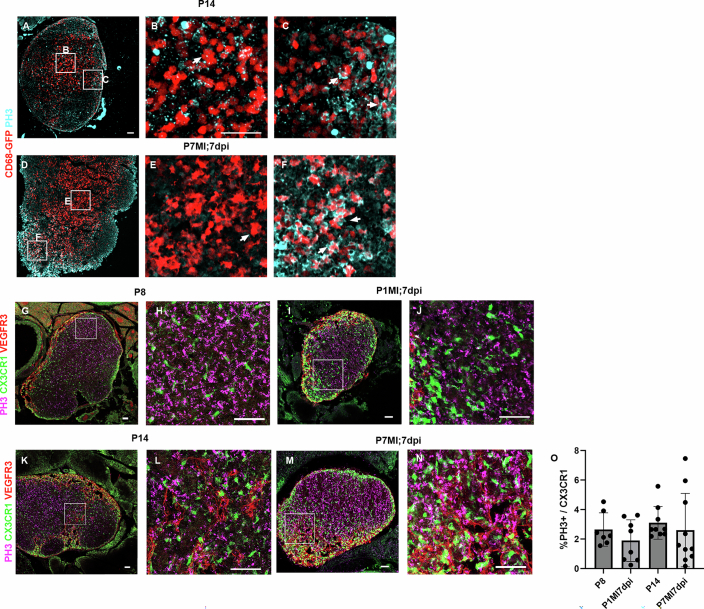
Proliferation of macrophages in the MLNs is restricted to the subcapsular sinus in both injured and intact post-natal mice. Visualisation of endogenous hCD68-eGFP and phospho-histone H3 (PH3) in MLNs from control (intact) P14 MLNs compared to MLNs from injured P7 mice at day 7 post-MI, revealed similar macrophage proliferation levels and distribution in both conditions (compare **A** to **D**). Macrophages were mostly negative for PH3 in the medullary sinus (**B** and **E**). Increased levels of PH3+ positive mitotic macrophages were found in the subcapsular sinus (**C**-**F**) in MLNs from both control and injured mice. Visualisation of endogenous CX3CR1-eGFP and PH3 in MLNs from uninjured mice at P8 and P14 compared to those from injured P1 and P7 mice 7 days post-MI (**D**-**N**). CD68+ and CX3CR1+ macrophages were similarly distributed and there was no significant difference in PH3+ macrophages between conditions (**O**). Data are presented as mean ± SEM; n = 7 for P8, n = 8 for P1MI7dpi, n = 9 for P14, 10 for P7MI7dpi. Significant differences were calculated using 1-way ANOVA followed by Tukey’s multiple comparisons test. Scale bars: 50 μm. Source data

To exclude the possibility that the increased CD68^+^ and CX3CR1^+^ macrophage numbers found in the MLNs after MI at P7 was due to a proliferative response of lymph-node-resident macrophages, MLNs were stained with the proliferation marker phospho-histone H3 (PH3; Extended Data Fig. 6a–o). Macrophages were found to proliferate in the subcapsular sinus of MLNs from both intact (P14) and P7 infarcted mice after 7 days (Extended Data Fig. 6c,f). However, most CD68^+^ and CX3CR1^+^ macrophages were localized to the medullary sinus and were negative for PH3 (Extended Data Fig. 6b,e,g–o). The absence of PH3^+^ macrophages from the medullary sinus of MLNs in mice after MI confirms that the elevated number of GFP^+^ macrophages from the adoptive transfer experiments arose from lymphatic-based clearance from the heart to the lymph nodes.

Taken together, these data reveal that cardiac lymphatics significantly clear CD68^+^, CX3CR1^+^ and F4/80^+^ macrophages via the afferent lymphatics to the draining MLNs after MI at P7 but do not clear macrophages from infarcted P1 hearts.

### Morphological changes in cardiac lymphatic endothelial cell–cell junctions at P1 versus P7

**Fig. 5:**
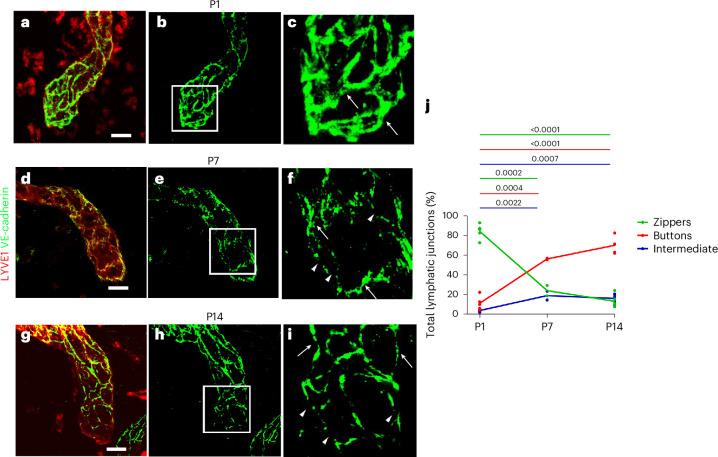
Cell–cell junctions of cardiac LECs undergo transformation during postnatal development. High-magnification confocal imaging of LECs within vessels stained for VE-cadherin and LYVE-1 enabled visualization of cell–cell junctions at different postnatal stages (**a**–**i**). The morphology of the junctions at P1 appeared to be continuous, resembling that of zippers (arrows in **c**). Zippered junctions were also observed at P7 (arrows in **f**), but there was also the emergence of discontinuous buttoned junctions (arrowheads in **f**) as well as those that were intermediate between zipper and button, indicative of a more cell-permeable endothelium. The more complete transformation to buttoned junctions was further evident by P14 (arrowheads in **i**), although some intermediate and zippered junctions were still evident at this stage (arrows in **i**). Quantification of the percent incidence of the three junction types (zippered, intermediate and buttoned) across the P1–P7–P14 timecourse (**j**) reveals the trend in transition from zippered (impermeable) to buttoned (permeable) during postnatal development. Macrophage morphology also transformed during this 2-week period. *n* = 5 for P1 and P14, *n* = 2 for P7; lymphatic vessel tips within the visual field were analyzed, 2–4 per heart. Significant differences were calculated using unpaired Student’s *t*-tests. Mean percent was plotted. Scale bars, 20 μm. Source data

A potential explanation underlying the differential ability of the cardiac lymphatics to traffic macrophages at P1 versus P7 after injury may arise from continued developmental changes to the lymphatic vasculature during the immediate neonatal period after birth. LECs have specialized intercellular junctions with different degrees of cell permeability, termed buttoned and zippered junctions^29^. In the lungs and trachea, the junctions of initial lymphatics undergo transformation during postnatal development, replacing tightly zippered with discontinuous, more cell-permeable button-like junctions, comprising mostly the same junctional proteins^30^. To study the junctions in initial lymphatics of the neonatal heart, we performed comparative immunostaining for VE-cadherin, whose expression is maintained in zippers, and LYVE-1 using high-resolution confocal imaging (Fig. 5). At P1, the initial lymphatics contained predominantly zipper-like junctions with continuous VE-cadherin expression and no gaps between neighboring LECs (Fig. 5a–c). By P7, button junctions were evident, defined as discontinuous approximately 3-μm gaps in VE-cadherin expression^29,31^, albeit still with a proportion of zippers (Fig. 5d–f); however, by P14, the junctions were predominantly button like, with discontinuous VE-cadherin and gaps between adjacent LECs (Fig. 5g–i), with the percent incidence of each junction type in Fig. 5j quantified as previously reported^31^. Thus, there is a transformation from zipper through intermediate to button junctions occurring between P1 and P7, which is still ongoing by P14, representing a dynamic change in junction morphology, which continues during later stages of postnatal heart development. The majority zipper junction phenotype at P1 would effectively exclude immune cell clearance after injury at this stage, whereas the appearance of button junctions by P14 corresponds to 7 days after MI at P7 and supports clearance at this stage, consistent with our adoptive transfer experiments (Fig. 4).

### Distinct molecular signatures and altered signaling between LECs and macrophages

**Fig. 6:**
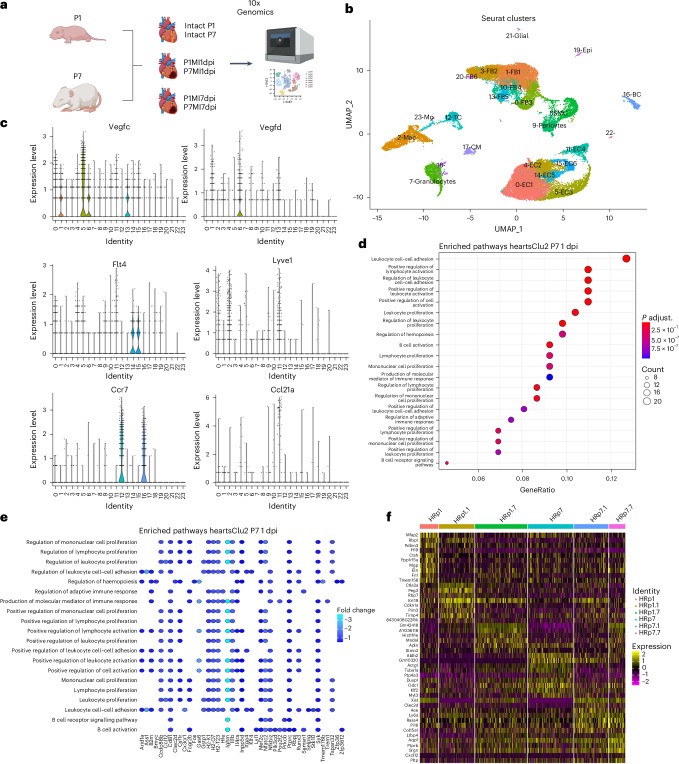
Expression of gene markers for macrophage and lymphatic endothelial cell populations. Schematic of the generation of scRNA-seq datasets of control and injured P1 versus P7 CD1 mice. Hearts were harvested at 1 day after MI (P1MI1dpi and P7MI1dpi) or 7 days after MI (P1MI7dpi and P7MI7dpi). For the intact conditions, the samples were collected at either P1 or P7 (Intact P1 and Intact P7). The samples were FACS sorted using 7-AAD to isolate live cells, and libraries were prepared for sequencing using the 10x Genomics platform (**a**). For each timepoint, one library was generated using pooled tissues dissected from three individual animals to control for differences among individual animals, surgery and tissue dissociation variations. UMAP plot showing the different major clusters, ‘heartsClu2’, in two dimensions (**b**). To validate the clustering, known lymphatic-associated and macrophage-associated genes were examined using the integrated scRNA-seq dataset (**c**). Unbiased Gene Ontology analysis identified pathways upregulated in macrophages after injury at P7 compared to P1 (**d**) and genes potentially driving these pathways (**e**). Significant differences between Gene Ontology term enrichment were calculated using Fisher’s exact test. **f**, Heatmap of changes in lymphatic endothelial cell gene expression across conditions. Panel **a** created with BioRender.com. Clusters: 0-EC1 (endothelial cells 1), 1-FB1 (fibroblasts 1), 2-Mac, 3-FB2 (fibroblasts 2), 4-EC2 (endothelial cells 2), 5-EC3 (endothelial cells 3), 6-FB3 (fibroblasts 3), 7-Granulocytes, 8-SMC (smooth muscle cells), 9-Pericytes, 10-FB4 (fibroblasts 4), 11-EC4 (endothelial cells 4), 12-TC (T cells), 13-FB5 (fibroblasts 5), 14-EC5 (endothelial cells 5), 15-EC6, 16-BC (B cells), 17-CM (cardiomyocytes), 18-unassigned, 19-Epi (epicardium), 20-FB6 (fibroblasts 6), 21-Glial (glial cells), 22-unassigned, 23-Mo (monocytes).

To understand whether differences observed in the lymphangiogenic response and immune clearance function of cardiac lymphatics after MI at P1 and P7 might also involve an altered LEC molecular phenotype and/or distinct signaling between LECs and macrophages, we carried out scRNA-seq at different timepoints using the 10x Genomics Chromium platform (Fig. 6a). Accordingly, the hearts from P1 and P7 CD1 mice were harvested 1 day and 7 days after MI, along with their corresponding non-infarcted controls, and the six individual cDNA libraries were constructed for scRNA-seq. The resulting data were analyzed in R using published Seurat pipelines with automated cluster annotation and manual consolidation of published gene markers^32,33^ for individual cell types.

**Extended Data Fig. 7:**
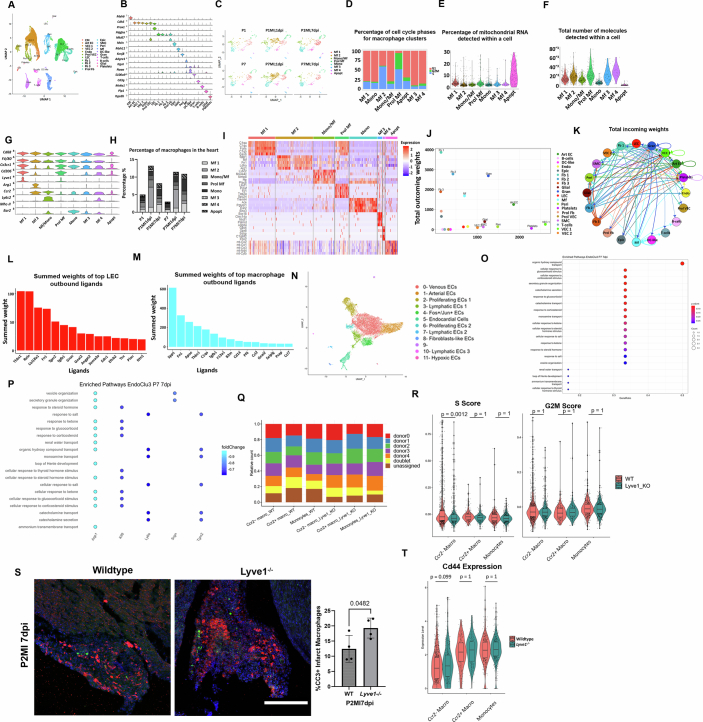
P1 versus P7 macrophage and monocyte populations have distinct proliferation profiles and differentially interact with lymphatic endothelial cells. Pooled UMAP plot showing the different major clusters (**A**). Stacked violin plots showing expression of marker genes for each cluster (**B**). UMAP of the macrophage and monocyte clusters separated based on timepoint and condition (P1 intact, P1MI1dpi, P1MI7dpi, P7 intact, P7MI1dpi and P7MI7dpi) (**C**). The percentage of cell cycle genes for each macrophage and monocyte cluster confirmed the existence of a proliferating population, with approximately half of the Mf2 cluster at phase S of cell division (**D**). The percentage of mitochondrial RNA (**E**) and the number of molecules (**F**) detected within cells confirmed the existence of an apoptotic population. Stacked violin plots showing expression of marker genes for each cluster suggests at least two tissue-resident macrophage populations, designated macrophage 1 (Mf1; *Lyve1*^*+*^*;Ccr2*^−^*;Arg1*^−^) and macrophage 2 (Mf2; *Lyve1*^−^*;Ccr2*^*+*^*;Arg1*^+^) (**G**). The relative percentage of each cell population at different timepoints and conditions as represented in a proportional bar chart (**H**). The unbiased differential expression analysis identified genes that were enriched in each cluster by comparing across all the other clusters (**I**). Comparison of total incoming path weights and total outgoing path weights across populations (**J**). Hierarchical network diagram of significant cell-cell interaction pathways, with arrows and edge colour indicating signalling direction ligand:receptor (**K**). Summed ligand weights across ligand and receptor target paths for top ligands in LECs (**L**) and macrophages (Mf) (**M**). Subclustering of endothelial cells to identify LECs (**N**). Unbiased GO term analysis showed pathways enriched at P7MI7dpi, vs P1MI7dpi but not between P1 and P7 (**O**). Genes implicated in enriched pathways (**P**). Relative abundance of macrophage subsets grouped by individual hearts following de-multiplexing of pooled samples (**Q**). Cumulative expression scores of cell cycle markers showed a decrease in *Lyve1*^−*/*−^ CCR2- macrophage proliferation, but no change in CCR2+ or monocytes (**R**). *In vivo* validation of increased apoptosis; CC3 co-expression with F4/80 within the infarct zone 7 days following MI at P2 was significantly increased in the *Lyve1*^−*/*−^ context (**S**). Violin plot indicating expression levels of CD44 were significantly reduced in the CCR2- macrophage cluster, but using vireo5^37^ were unchanged following loss of *Lyve1* (**T**). CM = cardiomyocytes, Art EC = arterial endothelial cells, VEC = venous endothelial cells, Endo = endocardium, Prol VEC = proliferating VEC, LEC = lymphatic endothelial cells, Fb = fibroblasts, Prol Fb = proliferating Fb, Epic = epicardium, SMC = smooth muscle cells, Peri = pericytes, Mf = macrophages, DC-like = dendritic cell-like, Gran = granulocytes. Data are presented as mean ± SD. Significant differences between Enriched Pathways in O and P were calculated using Fisher’s exact test. The box centre in violin plots in R and T indicates the median, the lower and upper hinges correspond to the first and third quartiles, and the whiskers extend to values with a distance from the hinges that is at most the inter-quartile range (IQR) multiplied by 1.5. Box plot parameters, including cell counts are available in Source Data. Bonferroni-corrected pairwise Wilcoxon Rank Sum test was used to determine significance in **R** and **T**. Unpaired Student’s t tests were used to determine significance in S. n = 4, 4 for S. Scale bar 200μm. Source data

The scRNA-seq data were analyzed to determine macrophage and monocyte heterogeneity in the macrophage populations as well as potentially altered signaling with LECs after MI at P1 versus P7 stages. Altogether, 23 clusters of cells with defined gene expression signatures were identified and visualized across two dimensions using uniform manifold approximation and projection (UMAP; Extended Data Fig. 7a). This revealed four major clusters: endothelial cells (which included a smaller subset of LECs), fibroblasts, immune cells as well as mural pericytes and smooth muscle cells as determined by canonical marker gene expression analyses (Extended Data Fig. 7b)

We initially sought to assess macrophage and monocyte heterogeneity in the designated macrophage population as well as potentially altered signaling with LECs after MI at P1 versus P7 stages. Using automated cluster annotation and manual consolidation of published gene markers for monocyte and macrophage subpopulations^32^, eight clusters with defined gene expression signatures were identified (Extended Data Fig. 7c). These included five macrophage clusters designated Mf1–Mf4, a proliferating macrophage cluster designated Prol Mf and two monocyte clusters designated Mono and Mono/Mf (Extended Data Fig. 7c–f). Next, we determined the representation of the various macrophage subpopulations in the postnatal heart (Extended Data Fig. 7g,h). In intact P1 and P7 hearts, macrophages and monocytes made up a combined 3–4% of non-myocyte cell types. There was a rapid expansion of Mf2 macrophages from 0.65% in the P1 control to 5.69% 1 day after P1 MI. Similarly, Mf2 macrophages increased from 0.22% in the P14 control to 5.27% 1 day after P7 MI, illustrating the immediate effect of injury on Mf2 expansion at both stages. A more gradual increase was observed in the percentage of Mf1 macrophages after MI: in the P1 control, 0.91% increased to 1.51% at 1 day after MI, finally reaching 2.26% at 7 dpi and 0.77% in the P7 control, rising to 1.24% at 1 day after MI and 2.59% at 7 days after MI. The percentage of Mf3 and Mf4 macrophages remained stable at 0.5% across all conditions and timepoints. The percentage of monocytes was low and similar in intact versus injured hearts at P1 (1.04% in intact controls versus 1.90% 7 days after MI), whereas they increased significantly with injury at P7 (from 0.7% in intact controls versus 3.39% at 7 days after MI). This suggests a limited contribution of monocytes after MI at P1, in contrast to increased infiltration after MI at P7. Finally, there was an increase in the percentage of Mono/Mf macrophages from 1.24% in the P1 control to 2.05% at 1 day after MI before it decreased to 0.99% at 7 days after MI. The increase observed in the percentage of Mono/Mf macrophages in P7 hearts after MI was more gradual, rising from 0.41% in the P7 control to 1.93% 7 days after MI (Extended Data Fig. 7h).

To distinguish whether the increase in the percentage of Mf1 and Mf2 macrophages observed after MI across early postnatal stages was due to proliferation or to monocyte recruitment and differentiation, the gene expression levels of the top five markers for Mf1, Mf2, Mono/Mf and Mono clusters were investigated in Prol Mf (Extended Data Fig. 7i). Prol Mf macrophages expressed high levels of Mf1 markers in all conditions and timepoints, whereas they expressed Mf2 markers mainly 1 day after MI at both P1 and P7. By contrast, there was little to no expression of Mono and Mono/Mf markers by Prol Mf macrophages. Absolute numbers of cells and differentially expressed genes are included in Supplementary Table 1.

Overall, these results indicate that postnatal hearts are populated predominantly by two tissue-resident macrophage populations: the Mf1 population that is *Lyve1*^+^;*Ccr2*^−^;*Arg1*^−^ and the Mf2 population that is *Lyve1*^−^;*Ccr2*^+^;*Arg1*^+^. These macrophage populations have a different temporal response to neonatal MI. Mf1 macrophages proliferate during postnatal development and expand gradually during the first 7 days after MI at P1 and P7, whereas Mf2 macrophages expand rapidly through proliferation 1 day after MI at P1 and P7 and subsequently decrease.

Macrophages have been described to interact with several cell populations in intact hearts and after injury^32,33^. In the present study, the macrophage interactome was analyzed using the scTalk pipeline in R^33^ to determine the expression of known ligands and receptors in all clusters of the postnatal heart. We subsequently focused on potential signaling between macrophages and LECs that might promote lymphangiogenesis and/or macrophage clearance in the postnatal heart. LECs showed substantially more inbound connections than outbound (Extended Data Fig. 7j,k). By contrast, macrophages were found to have significantly more outbound connections (Extended Data Fig. 7j,k), communicating with endothelial cells, platelets, pericytes and all immune cells by paracrine signaling, with evidence of additional autocrine signaling (Extended Data Fig. 7k). LECs appeared to communicate primarily with fibroblasts, granulocytes and platelet cells (Extended Data Fig. 7k). Further analysis of ligands secreted by LECs and macrophages (Extended Data Fig. 7l,m) revealed that *Reln* was the most prevalent secreted ligand from LECs, with *Angpt2* (angiopoietin-2) and *Efnb2* (ephrin-B2) as well as *Sema3a* (semaphorin-3A), *Tnc* (tenascin C) and *Ntn1* (netrin-1) also among the top outbound ligands (Extended Data Fig. 7l). RELN has been described as cardioprotective after MI^6,23^ and more recently as a lymphangiocrine signal important for cardiomyocyte homeostasis and efficient heart repair and function after neonatal mouse MI^23^, whereas ANGPT2 and EFNB2 have angiogenic and lymphangiogenic functions^34,35^. As anticipated, ligands found to be expressed by macrophages were categorized based on immune response-related functions; for example, *Ccl7* (C-C motif chemokine ligand 7) has anti-inflammatory functions, and *Ccl2* (chemokine C-C motif ligand 2) is involved in the migration of monocytes and macrophages (Extended Data Fig. 7m).

To conduct pathway enrichment analysis, we reclustered our scRNA-seq data to obtain pooled macrophage and LEC groups for each condition (Fig. 6b). To validate our reclustering, we investigated genes of interest (Fig. 6c). The expression of *Vegfc* and *Vegfd* was negligible in macrophages and LECs, which likely accounts for the lack of lymphangiogenesis at P1 (Fig. 2e) despite high macrophage accumulation after MI. Of note, *Vegfc/d* is not expressed by embryonic macrophages but is expressed by adult macrophages^3^. We found the expression of *Lyve1* mainly in macrophages and LECs, as expected. The expression of *Flt4* (encoding VEGFR3) was elevated in LECs with low-level expression in some vascular endothelial cells, likely indicating a common venous origin. Finally, we investigated the expression of the dendritic cell marker *Ccr7* and its ligand *Ccl21*. We found that our macrophage cluster expressed negligible levels of both, whereas LECs express *Ccl21* at high levels, consistent with our qPCR analysis (Extended Data Fig. 4g). This, in turn, suggested that dendritic-cell-like populations were essentially undetectable in the injured hearts across conditions, such that any inference drawn from our trafficking experiments with CD68^+^ cells can be exclusively applied to macrophages at the observed timepoints, as we previously validated by F4/80 staining of the CD68^+^ trafficked population (Fig. 4n). Subsequently, pathway enrichment analysis of macrophages was conducted and revealed upregulation of leukocyte adhesion, activation and proliferation after injury at P7 compared to P1 (Fig. 6d,e).

Reclustering our LECs (Extended Data Fig. 7n) facilitated in-depth unbiased differential gene expression analysis for LECs (Fig. 6f), which revealed distinct molecular signatures between LECs at P1 versus P7, with no enriched pathways in the uninjured state, but enrichment of several pathways and associated genes at P7 relative to P1 after injury (Extended Data Fig. 7o,p).

**Extended Data Fig. 8:**
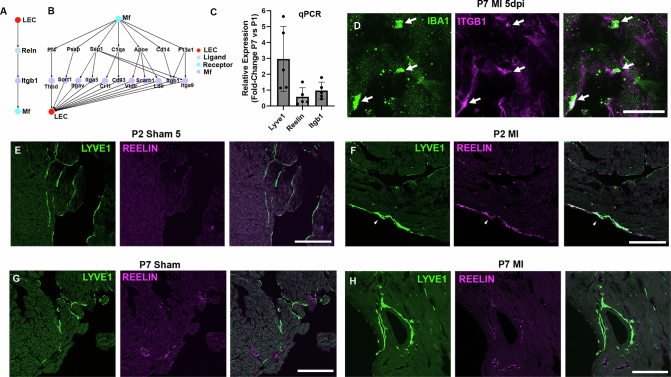
Network analysis showing LEC-macrophage signalling. Tree plot showing outgoing connections from the LECs to Mf (**A**) and from Mf to LECs (**B**). The single outgoing LEC signal was identified as Reelin (Reln), a previously described lymphangiocrine factor, interacting with ITGβ▢ on Mf (**A**). Top node refers to source population, second layer to ligands, third layer to receptors and bottom node representing the target population (**A**, **B**). *Reln* expression was shown by qPCR to decrease between P1 (n = 6) and P7 (n = 5), while *Itgb1* expression was unchanged (**C**). Immunostaining for ITGFB1 combined with IBA1 (**D**) and REELIN combined with LYVE-1 (**E**-**H**) in resident macrophages and LECs, respectively, revealed elevated expression of Reelin at P2MI5dpi compared to P7MI5dpi and sham controls across equivalent timepoints (Figure **E**-**H**). Data are presented as mean ± SD. Qualitative observations in **D**-**H** were seen across tissue sections and in 1 additional heart. Scale bar: 25 μm for D; 150 μm for **E**-**H**. Source data

Finally, potential interactions between ligands and receptors expressed by LECs and macrophages were examined (Extended Data Fig. 8a,b). The scTalk analysis revealed only a single potential interaction between the LEC ligand RELN and the macrophage receptor integrin β1 (ITGB1), which has not previously been described in these cell populations. Subsequent qPCR analyses suggested that *Reln* was downregulated at P7 compared to P1, whereas *Itgb1* expression was unchanged (Extended Data Fig. 8c). We confirmed the qPCR data at the level of protein expression by immunostaining for REELIN combined with LYVE-1, ITGB1 and IBA1. Here, we examined LECs at 5 days after MI following surgery at P2, to be consistent with the stages examined in the prior study^23^, compared to 5 days after MI at P7 and uninjured controls. This revealed positive staining of ITGB1 in IBA1^+^ macrophages (Extended Data Fig. 8d) and elevated expression of REELIN in LECs at P2 compared to P7 at 5 days after MI (compare Extended Data Fig. 8e,f to Extended Data Fig. 8g,h), consistent with a role in the early postnatal regenerative response and validating the scRNA-seq pathway analyses (Extended Data Fig. 8a).

### LYVE-1 plays a role in the regenerative response to injury at P1

**Fig. 7:**
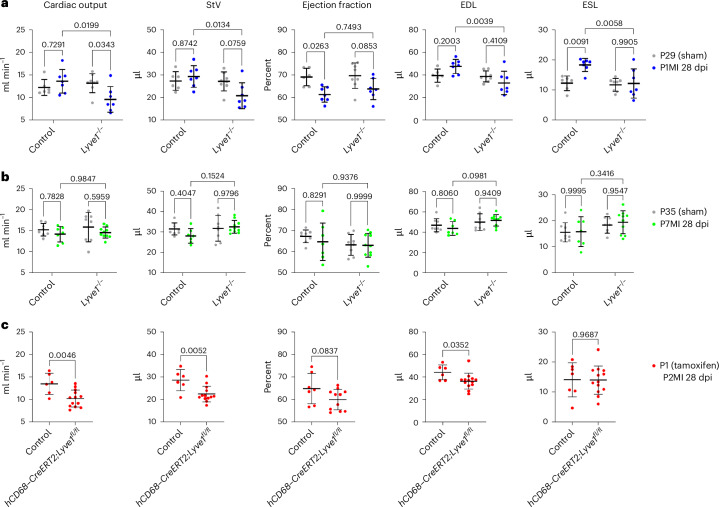
Functional MRI parameters of P1 versus P7 *Lyve1* KO and macrophage-specific *Lyve1* KO hearts 28 days after MI. Plots from longitudinal cine MRI performed on *Lyve1*^*+/*−^ and *Lyve1*^−*/*−^ mice 28 days after MI at P1 and P7 as well as in intact control littermates at comparable P29 and P35 stages (**a**,**b**). MRI revealed significantly reduced cardiac output in P1 *Lyve1*^−*/*−^ mice at 28 days after MI compared to P29 intact *Lyve1*^−*/*−^ controls (**a**). The reduced cardiac output was even more significant than reductions observed for *Lyve1*^−*/*−^ mice injured at P7 at 28 days after MI compared to P35 intact *Lyve1*^−*/*−^ controls (**b**). Plots from MRI performed on *hCD68–CreERT2;Lyve1*^*flox/flox*^ mice 28 days after MI at P2 reveal impaired functional recovery across cardiac output, StV, ejection fraction and end diastolic volume consistent with **a**, differing only in end systolic volume (**c**). Data are presented as mean ± s.d. *n* = 6 for P29 control, *n* = 7 for P1MI28dpi control, *n* = 8 for P29 *Lyve1*^−*/*−^, *n* = 7 for P1MI28dpi *Lyve1*^−*/*−^, *n* = 8 for P35 control, *n* = 7 for P7MI28dpi control, *n* = 8 for P35 *Lyve1*^−*/*−^, *n* = 10 for P7MI28dpi *Lyve1*^−*/*−^, *n* = 7 for P2MI28dpi, *n* = 13 for P2MI28dpi. Significant differences were calculated using two-way ANOVA for **a** and **b** and unpaired two-tailed Student’s *t*-test for **c**. Source data

**Extended Data Fig. 9:**
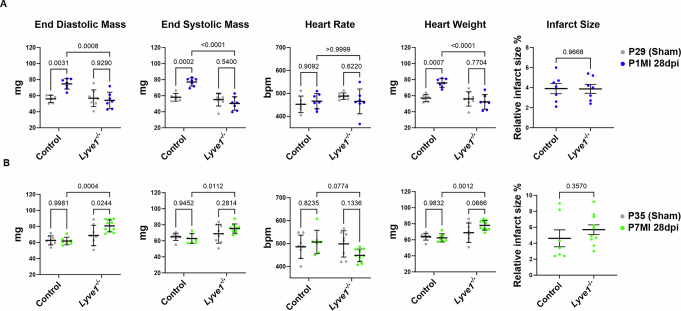
Additional functional MRI parameters of Lyve1 KO hearts 28-days after MI at P1 and P7. Plots from longitudinal cine MRI performed on *Lyve1*^*+/*−^ and *Lyve1*^−*/*−^ mice 28 days after MI at P1 and P7, as well as in intact control littermates at comparable P29 and P35 stages (**A**,**B**). MRI revealed significantly reduced end diastolic mass (EDM), end systolic mass (ESM), and heart weight (hwt) in P1 *Lyve1*^−*/*−^ mice at 28 days post-MI compared to *Lyve*^*+/*−^ MI controls at the equivalent timepoint (**A**). Conversely, EDM, ESM, and hwt were all significantly increased in *Lyve1*^−*/*−^ mice injured at P7 at 28-days post-MI compared to *Lyve1*^*+/*−^ 28dpi controls (**B**). Data are presented as mean ± SD; n = 6 for P29 control, n = 7 for P1MI28dpi control, n = 8 for P29 *Lyve1*^−*/*−^ n = 7 for P1MI28dpi *Lyve1*^−*/*−^. n = 8 for P35 control, n = 7 for P7MI28dpi control, n = 8 for P35 *Lyve1*^−*/*−^ n = 10 for P7MI28dpi *Lyve1*^−*/*−^. Significant differences were calculated using 2-way ANOVA. Source data

In addition to an unbiased analysis of putative regulators of LEC–macrophage interactions, we also took a candidate approach focused on the lymphatic endothelial receptor LYVE-1, given its expression by both cardiac lymphatics and macrophages during early and later postnatal stages, as shown in Fig. 5. As we previously reported, global KO of *Lyve1* leads to disruption of cardiac macrophage clearance after MI in adult mice, attributable to an LEC-autonomous defect that results in augmented pathological remodeling, increased fibrosis and impaired cardiac function^3^. Macrophages are essential for cardiac regeneration after MI at P1 (ref. ^21^), and, as shown here, macrophages are cleared less efficiently after MI at P1 compared to P7. Consequently, we hypothesized that deletion of *Lyve1* would not affect resident macrophage clearance in P1 injured hearts during regeneration but would negatively impact on the clearance of inflammatory/pro-fibrotic macrophages during repair at P7 (as in adults^3^). To test this hypothesis, initially we studied global *Lyve1* KO mice comparing *Lyve1*^*+/*−^ and *Lyve1*^−*/*−^ animals after MI to include functional longitudinal imaging by MRI on day 28 after MI at P1 or P7 (P1MI28dpi and P7MI28dpi, respectively). Intact *Lyve1*^*+/*−^ and *Lyve1*^−*/*−^ mice were also scanned at day 29 or day 35 as controls for P1MI28dpi and P7MI28dpi, respectively. Left ventricular function was assessed by measuring the cardiac output, stroke volume (StV), ejection fraction, end diastolic lumen (EDL) and end systolic lumen (ESL) (Fig. 7a,b). Other cardiac parameters were also calculated: end systolic mass (ESM), end diastolic mass (EDM), heart weight (hwt), heart rate and relative infarct size (Extended Data Fig. 9). Surprisingly, we observed impaired cardiac function in *Lyve1*^*+/*−^ and *Lyve1*^−*/*−^ mice after MI at P1 compared to their respective intact controls (Fig. 7a). Specifically, ejection fraction was significantly decreased 28 days after MI at P1 in *Lyve1*^*+/*−^ mice compared to intact *Lyve1*^*+/*−^ P29 (*P* = 0.026; Fig. 7a). Cardiac output was significantly reduced 28 days after MI at P1 in *Lyve1*^−*/*−^ mice compared to intact *Lyve1*^−*/*−^ P29 (*P* = 0.034; Fig. 7a). Also, StV was significantly lower 28 days after MI at P1 in *Lyve1*^−*/*−^ mice compared to the same condition in *Lyve1*^*+/*−^ (*P* = 0.0134; Fig. 7a). By contrast, due to inherent variation in size and response to injury, no statistically significant differences were observed in ejection fraction, cardiac output, ESL, EDL or StV between the different conditions and timepoints after injury at P7 (Fig. 7b). The values of ESM, EDM and hwt were found to be statistically significant after MI at P1 in *Lyve1*^*+/*−^ mice compared to the other P1 conditions as well as after MI at P7 in *Lyve1*^−*/*−^ mice compared to *Lyve1*^*+/*−^ mice (Extended Data Fig. 9). The heart rate was similar in all conditions examined (Extended Data Fig. 9). Interestingly, a trend for larger relative infarct size was detected 28 days after MI at P7 in *Lyve1*^−*/*−^ mice compared to the other conditions; however, this was not statistically significant (Extended Data Fig. 9).

Taken together, these studies revealed an unexpected association between *Lyve1* and functional outcome after MI at P1, a timepoint normally associated with complete regeneration and preserved function^14^. This confounded our initial hypothesis that, given that pro-regenerative macrophage clearance is not occurring at P1, loss of LYVE-1 would have no effect on recovery from early postnatal MI.

### LYVE-1 maintains the pro-regenerative phenotype of tissue-resident macrophages

**Extended Data Fig. 10:**
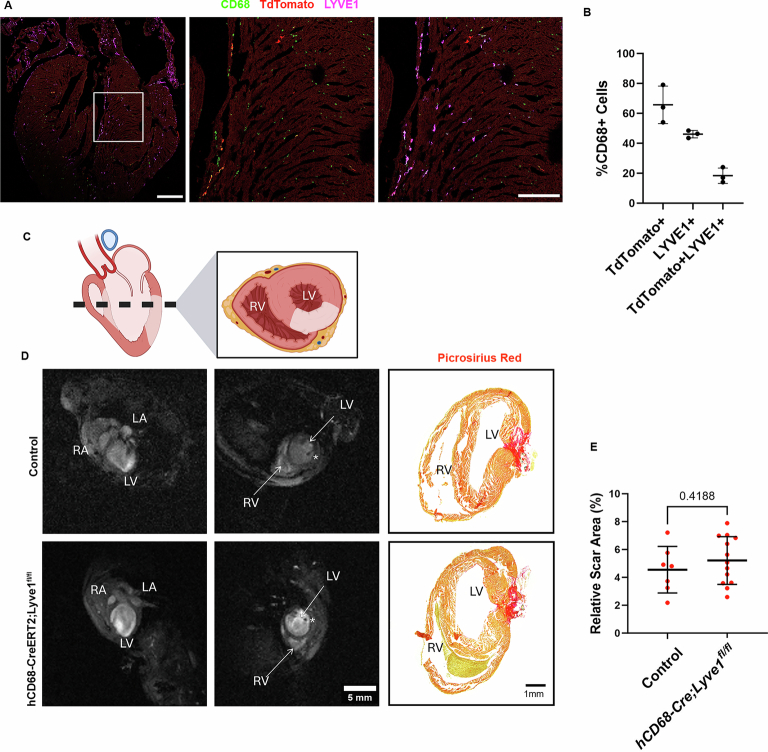
*hCD68-CreERT2;R26R-TdTomato* reports LYVE-1 positive macrophages in P2 hearts 7 days post-MI and MRI cine images of *hCD68-CreERT2;Lyve1*^*fl*^*°*^*x/fl*^*°*^*x*^ hearts 28-days post-MI with corresponding picrosirius red staining. Macrophages expressing TdTomato in P2 infarcted hearts at 7dpi. LYVE-1 + /TdTomato+ macrophages confirmed by co-staining with CD68 and LYVE-1 (**A**). Quantification of co-staining in sections (n = 3) (**B**). Tamoxifen injected at P1, MI surgery at P2 to avoid inducing respiratory failure. Longitudinal and transverse cine MRI representative images from control and *hCD68-CreERT2*+ animals at P2MI28dpi accompanied by transverse picrosirius sections matched to the corresponding level and illustrated by schematic (**C**, **D**). Picrosirius red staining revealed no significant difference in relative scar area between control and *hCD68-CreERT2*+ animals at 28 days post-MI (**E**). Asterisk marks suture site. RA = right atrium, LA = left atrium, RV = right ventricle, LV = left ventricle. n = 7 for control, n = 13 for *hCD68Cre*^*ERT2*^*;Lyve1*^*fl/fl*^ hearts. Panel **C** created in BioRender; Chapman, B. (2025) https://BioRender.com/z1nstjj. Unpaired Student’s t test was used to determine significance. Data are presented as mean ± SD. In A, scale bar 350μm; zoomed 150μm. In D, scale bar 5 mm in MRI; 1 mm in picrosirius sections. Source data

The *Lyve1* global KO phenotype suggested an additional, unanticipated role for LYVE-1 outside of the lymphatic endothelium and immune cell clearance and especially given its known expression in tissue-resident macrophages, which are essential for regeneration^21^ and are retained rather than cleared from injured P1 hearts. Accordingly, we hypothesized that LYVE-1 in resident cardiac macrophages (Fig. 6) may play an active role in maintaining their pro-regenerative phenotype. To address this possibility, we conditionally deleted *Lyve1* in macrophages by crossing a newly derived *Lyve1-floxed* line (Jackson, D. G., unpublished) with the recently described *hCD68–CreERT2* mouse line that effectively targets resident macrophages across tissues^36^. We initially tested for appropriate Cre-driver activity by crossing *hCD68–CreERT2* mice with a *R26R–tdTomato* reporter line, which revealed tdTomato reporter expression in CD68^+^LYVE-1^+^ resident macrophages up to 7 days after tamoxifen administration at P1 and MI at P2 (Extended Data Fig. 10a,b). Of note, tamoxifen and surgery cannot be carried out on the same day as the tamoxifen induces respiratory failure, thereby necessitating pretreatment (at P1) followed by surgery 1 day later (at P2).

**Fig. 8:**
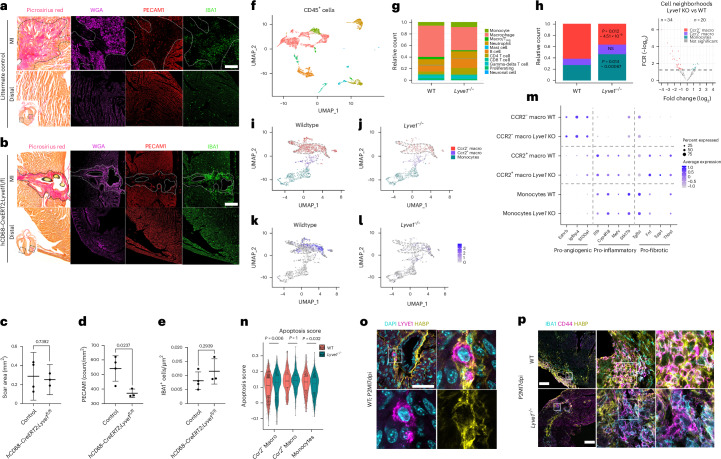
Impaired vascular response in *hCD68*–*CreERT2;Lyve1*^*flox/flox*^ infarcted hearts and a population shift toward inflammatory monocytes in *Lyve1* KO CD45^+^ cells at day 7 after MI. Identification of scar area at 7 dpi using wheat germ agglutinin (WGA) and Picrosirius red fibrosis stain. Visualization of vasculature and macrophages with CD31 (PECAM1) and IBA1, respectively. Representative images of littermate control (**a**) and *hCD68–CreERT2;Lyve1*^*flox/flox*^ (**b**) sections illustrating reduced neovascular response in *Cre*^+^ sections. Quantification of scar area revealed no significant difference between conditions (**c**). Quantification of discrete PECAM1 signal within the infarct zone relative to area as an indicator of vascular response revealed significantly reduced PECAM1-stained vasculature in *Cre*^+^ hearts (**d**). Quantification of macrophages by IBA1 stain revealed no significant difference between conditions (**e**). scRNA-seq was conducted, comparing CD45^+^ enriched cells from neonatal *Lyve1* KO versus WT hearts at P2MI7dpi. The samples were FACS sorted using 7-AAD and CD45 to isolate live CD45^+^ cells, and libraries were prepared for sequencing using the 10x Genomics platform. UMAP plot of grouped WT and *Lyve1* KO CD45^+^ cells (**f**). Comparison of CD45^+^ cell subset proportions between WT and *Lyve1* KO conditions (**g**), including statistical analyses of quantified differences between subsets after deconvolution of individual heart samples by Vireo5 (ref. ^37^) (**h**). Macrophage subset clustering in WT (**i**) and *Lyve1* KO (**j**). *Lyve1* gene expression within subclusters in WT (**k**) and *Lyve1* KO (**l**). Dot plot illustrating relative expression of key pro-angiogenic, pro-inflammatory and pro-fibrotic genes in each macrophage subset and between WT and *Lyve1* KO (**m**). Cumulative apoptotic marker expression scores between conditions and macrophage subsets (**n**). Representative LYVE1^+^ macrophage possessing an HA glycocalyx (**o**). Differential HA glycocalyx staining between WT and *Lyve1*^−*/*−^ macrophages (**p**). *n* = 4 control hearts, *n* = 3 *hCD68–CreERT2;Lyve1*^*flox/flox*^ hearts; four sections per heart for **a**–**e**, pooled samples from *n* = 5; five hearts for **f**–**m**. The box center in violin plots in **n** indicates the median; the lower and upper hinges correspond to the first and third quartiles; and the whiskers extend to values with a distance from the hinges that is at most the interquartile range multiplied by 1.5. Box plot parameters, including cell counts, are available in the Source Data. Unpaired Student’s *t*-tests were used to determine significance in **c**–**e**. Bonferroni-corrected pairwise Wilcoxon rank-sum test was used to determine significance in **n**. Qualitative observations in **o** and **p** were repeated across the scar and in a second infarcted heart. Scale bars, 200 μm for **a**, **b** and **p**; 50 μm for **o**. FDR, false discovery rate; HA, hyaluronic acid; macro, macrophage; NS, not significant; T_reg_, regulatory T cell. Source data

We next assessed functional parameters by MRI of macrophage-specific *Lyve1* KO animals compared to littermate controls 28 days after MI (Fig. 7c and Extended Data Fig. 10c–e). This revealed impaired function with significantly reduced cardiac output (*P* = 0.0046) in the *hCD68–CreERT2;Lyve1*^*flo*^^*x/flo*^^*x*^ P2 infarcted hearts at day 28 (Fig. 7c). StV and end diastolic volume (Fig. 7c) were also decreased significantly (*P* = 0.0052 and *P* = 0.0352, respectively), consistent with what we observed in the global *Lyve1* KO animals P1MI (Fig. 7a). To gain insight into the cellular mechanism(s) underpinning the change in cardiac function after neonatal MI, we carried out Picrosirius red staining, which suggested a reduced fibrotic area between *hCD68–CreERT2;Lyve1*^*flox/flox*^ P2 infarcted hearts compared to their littermate controls; however, this was not significant at 7 dpi or 28 dpi (Fig. 8a–c and Extended Data Fig. 10e). Given the acknowledged role of macrophages in infarct neovascularization, we conducted further histological analyses with CD31/PECAM immunostaining, in line with previous work^21^. Intriguingly, this revealed an impaired vascular response at day 7 after MI in the macrophage-specific mutants after MI (Fig. 8d). No significant differences were observed in macrophage concentration at the infarct site (Fig. 8e).

To interrogate the molecular role of LYVE-1 in macrophages, scRNA-seq analysis of pooled CD45^+^ enriched cells from neonatal *Lyve1* KO versus WT hearts (*n* = 5 per group) at P2MI7dpi was conducted, thus capturing all immune cells including macrophages at a timepoint consistent with the observed impaired neovascularization in *hCD68–CreERT2;Lyve1*^*flox/flox*^ animals (Fig. 8f–m). To facilitate statistical analysis of pooled samples, deconvolution of individuals by unique single-nucleotide polymorphisms per heart was carried out using Vireo5 (ref. ^37^) (Extended Data Fig. 7q). Initial clustering identified macrophage subsets, grouped broadly as monocytes, CCR2^+^ macrophages and CCR2^−^ macrophages (Fig. 8f). We observed statistically significant reductions in the CCR2^−^ subpopulation cell neighborhoods (*P* = 0.012–4.51 × 10^−9^) and corresponding statistically significant elevation of the CCR2^+^ subpopulation in the *Lyve1*^−/−^ samples compared to WT (*P* = 0.014–0.00067) (Fig. 8g,h and Supplementary Table 2). This represents a loss of the CCR2^−^ tissue-resident macrophage population, which is replaced by CCR2^+^ monocyte-derived populations in the KO background. Notably, the *Lyve1*^+^ macrophage clusters identified in WT hearts were still present in the KO setting, albeit in significantly smaller numbers and without expressing *Lyve1*, confirming that loss of *Lyve1* does not result in complete loss of this subpopulation (Fig. 8i–l). Furthermore, unbiased differential gene expression analysis demonstrated alignment of our three identified populations (monocytes, CCR2^+^ and CCR2^−^) with previously reported signatures in terms of effects on angiogenesis, fibrosis and inflammation (Fig. 8m). These gene expression profiles were unchanged between WT and KO conditions (Fig. 8m), strengthening the likelihood that the observed phenotype in the *Lyve1* KO background is due to an effect on the reduced CCR2^−^ macrophage population, leading to increased inflammatory monocyte recruitment.

The reduction in CCR2^−^ macrophages was accompanied by a statistically significant increase in apoptotic marker gene expression (Fig. 8n) and decreased proliferative marker gene expression within this subset (Extended Data Fig. 7r). In CCR2^+^ macrophage and monocyte populations, there was no change in apoptotic or proliferative score (Fig. 8n and Extended Data Fig. 7r). Increased tissue-resident macrophage apoptosis was validated in vivo via staining WT and *Lyve1* KO hearts for F4/80 and the apoptosis marker cleaved caspase 3 (CC3) at 7 days after MI at P2, matching the timepoint of the single-cell dataset. Staining revealed a significant increase in the number of cells co-expressing CC3 and F4/80 in KO hearts after MI (Extended Data Fig. 7s).

To investigate the mechanism by which loss of *Lyve1* predisposes CCR2^−^ macrophages to apoptosis, we considered the known role of the closely related LYVE1 homologue CD44 in dendritic cells and macrophage populations, where it anchors the hyaluronic acid glycocalyx. It is known that loss of *Cd44* disrupts the dendritic cell glycocalyx and predisposes the cell to apoptosis by revealing pro-apoptotic elements on its surface^9,38^. Our scRNA-seq analysis revealed that *Cd44* expression was significantly lower in the CCR2^−^ macrophage population compared to CCR2^+^ macrophages and monocytes, suggesting that LYVE-1 may substitute for CD44 in this subpopulation (Extended Data Fig. 7t). We, therefore, carried out immunostaining for hyaluronic acid binding protein (HABP) to determine the presence of a hyaluronic acid glycocalyx in LYVE-1^+^ macrophages in WT mice 7 days after MI at P2 (Fig. 8p). Subsequently, we examined representative images of macrophages between WT and *Lyve1*^−*/*−^ hearts at 7 days after MI at P2 and identified an apparent loss and/or notable reduction of glycocalyx in a proportion of *Lyve1*^−*/*−^ macrophages (Fig. 8p). This suggests that tissue-resident macrophages may be predisposed to apoptosis in the absence of LYVE-1, as previously described for dendritic cells in the context of CD44 (refs. ^9,38^).

Collectively, these data reveal a role for LYVE-1 in the survival and maintenance of tissue-resident macrophages during the regenerative response to heart injury.

## Discussion

Although the cardiac lymphatics have received considerable attention to date, studies have largely focused on their developmental origins and the adult lymphangiogenic response to heart injury across model organisms^1–3,25^. There has been no prior investigation of the function of the cardiac lymphatics during neonatal heart regeneration and the transition to fibrotic repair within the first week of life.

We observed that the cardiac lymphatics continue to grow significantly during the initial 2 weeks after birth in mice—first across the dorsal surface and then by ventral surface expansion, reaching full length and density by P16 and P28 on the dorsal and ventral surfaces, respectively. It is possible that this temporal discrepancy arises from the heterogeneous origins of cardiac LECs. The lymphatics of the heart have been described to emerge and grow not only through sprouting of preexisting vessels (lymphangiogenesis) but also from isolated LECs (lymphvasculogenesis), as reported for zebrafish and mice, and from a variety of sources including hemogenic endothelium, second heart field progenitors and paraxial mesoderm (reviewed in ref. ^39^). Here, our observation of short, isolated lymphatic vessels present mainly on the dorsal surface during early postnatal stages suggests a further undefined non-venous source^25,40,41^.

In adult mammals, the lymphatic vasculature is compromised near the site of injury (infarct region), resulting in increased edema and reduced ability to clear immune cells. Although lymphatics grow and sprout through lymphangiogenesis after MI in adult mice, this endogenous response is insufficient to optimize repair and preserve cardiac function^1,2^. Several gain-of-function studies have used the ligand VEGFC to enhance lymphangiogenesis after MI in adult mice and have reported improved clearance of interstitial fluids and immune cells, most notably macrophages, consequently improving pathological remodeling of the heart and function^1–4^. Interestingly, a previous study reported that blocking endogenous lymphangiogenesis, through VEGFR3 or VEGFC/D loss of function, does not lead to increased edema or impaired cardiac function after MI^42^. However, this loss-of-function study targeted endogenous lymphangiogenesis, which, in itself, is suboptimal, and did not investigate the effects of gain of function to promote enhanced lymphangiogenesis, as previously demonstrated^1–4^. Collectively, these studies support the hypothesis that the endogenous response of cardiac lymphatics is insufficient to influence outcome after MI, whereas an augmented lymphatic response, through the administration of exogenous growth factors, improves repair and function.

In contrast to adult mammals (including humans), it is well established that zebrafish^43,44^, the surface-dwelling Mexican cavefish^45^, neonatal mice and human infants can regenerate their hearts after injury^16,17,46^. In zebrafish, cryoinjury stimulates cardiac lymphatic growth at the site of injury^25,47^, and disruption of vegfc–vegfr3 signaling impairs the lymphatic response and associated clearance of infiltrating immune cells, leading to prolonged inflammation, persistent scarring and reduced regeneration^48^. In the presence of an intact lymphatic network, scarring in the adult zebrafish heart is transient, whereas it is more permanent in the adult mouse heart.

Functional cardiac lymphatics are required to maintain an optimal immune cell load after adult heart injury, by providing conduits for efficient clearance of immune cells (neutrophils, macrophages, dendritic cells and T cells) to draining MLNs^3^. Macrophages dominate the acute response to MI in terms of sheer numbers derived from infiltrating monocytes, which are recruited from bone marrow and splenic reservoirs, with an initial pro-inflammatory (CCR2^+^Ly-6C^high^) phenotype, which then gives rise to pro-reparative (Ly-6C^low^) macrophages in situ^49^. The infiltrating monocyte-derived macrophages are thought to replace a tissue-resident macrophage population (defined as CCR2^−^Ly-6C^−^) during the initial acute response to injury. The timing of lymphatic clearance in adult infarcted hearts appears to coincide with the initial pro-inflammatory phase but impacts on downstream repair, suggesting a continuum of monocyte–macrophage function across the acute stages of heart injury^3^. By contrast, in neonatal mice after MI, tissue-resident macrophages increase in number without infiltration of monocytes from remote sources^18–20^, and general depletion of resident macrophages by clodronate liposome treatment inhibits cardiac regeneration and leads to reduced cardiac function after MI at P1 (ref. ^21^). Thus, we reasoned that pro-regenerative macrophages would not need to be cleared by the cardiac lymphatics after injury at P1, whereas, at P7, when macrophages contribute to fibrotic repair, there would need to be trafficking, analogous to that in the injured adult heart^3^. This was suggested by a significantly reduced lymphatic response 7 days after MI at P1 compared to P7—an observation that was background dependent. The difference was most pronounced in C57BL/6J mice, whereas, in CD1 mice, there was a greater lymphangiogenic response at P1 in response to injury, albeit still reduced compared to P7. We confirmed impaired trafficking at P1 by adoptive transfer of adult splenic hCD68–eGFP^+^ macrophages into WT infarcted neonatal recipient hearts followed by analysis of the recipient MLNs. Here, the adult donor-sourced labeled macrophages were competent for trafficking and clearance, as we previously demonstrated^3^. Viable GFP^+^ macrophages were detected in recipient hearts, confirming appropriate transfer, but were observed only in the draining MLNs of mice at P7 and not at P1. We validated the capacity of macrophages for trafficking at P7 via MLN imaging in *hCD68–eGFP* mice after MI and demonstrated that this was absent after injury at P1. Given that the macrophage response to neonatal MI comprises a large number of tissue-resident macrophages, we imaged *CX3CR1–eGFP* mouse MLNs to confirm that CX3CR1^+^ tissue-resident macrophages are also trafficked after injury at P7 but not at P1.

Impaired lymphatic clearance at P1 may be the result of several emergent factors during the postnatal period, including maturation of the lymphatic vessels to a state permissive for macrophage ingress and clearance, differences in signaling between LECs and macrophages and/or temporal functional requirements for essential candidate facilitators of lymphatic trafficking, such as LYVE-1. The LECs that form lymphatic vessels are interconnected by specialized cell–cell junctions, with previous studies describing a transition from impermeable zipper-like to cell-permeable buttoned junctions during embryonic development, as evidenced in the mouse trachea, diaphragm and lungs^29,50,51^. This transition starts at approximately embyonic day 17.5 and is completed by P14 (ref. ^30^). We observed a similar process in the early postnatal heart with zippered junctions predominating in the initial lymphatic vessels during the first days after birth and buttoned junctions appearing during the second week of life. This maturation of the initial lymphatics likely affects their ability to clear immune cells from the heart to lymph nodes at early neonatal (P1) stages. At P7, when clearance was evident from our adoptive transfer experiments, there was still an incidence of zippered junctions, suggesting an ongoing developmental process through to adulthood when all junctions are button like and the lymphatic endothelium is relatively cell permeable^3^. To investigate whether temporal alterations in signaling between the LECs and macrophages might influence clearance of the latter, we carried out scRNA-seq on P1 versus P7 infarcted hearts at days 1 and 7 after injury. Whereas multiple signals from macrophages to LECs were identified, the reciprocal signaling was remarkably restricted to only a single pathway that was elevated at P1 versus P7 after injury. We observed that the extracellular matrix glycoprotein RELN was expressed in LECs at high levels at P1 compared to P7, and pathway analyses identified a potential interaction with ITGB1 on macrophages. In a recent study, RELN was identified as a lymphangiocrine signal important for cardiomyocyte homeostasis and efficient heart repair and function after neonatal mouse MI^23^. Moreover, RELN has been identified in LECs in human fetal hearts, adding potential relevance to human physiology and disease^52^. Neither the target cell type(s) nor relevant receptor(s) were previously identified, and it will be of interest in future studies to functionally assess the RELN–ITGB1 pathway in LECs and macrophages correlated with heart regeneration. Finally, we investigated a role for LYVE-1 acting across the P1–P7 regenerative window, given its pivotal role in the hyaluronic-acid-mediated adhesion and entry of immune cells to initial lymphatics^8^. We hypothesized that global *Lyve1* gene deletion would have no effect on P1 heart regeneration given that macrophage trafficking is negligible at this stage, consistent with the need to retain the pro-regenerative tissue-resident population in situ^21^. Unexpectedly, MRI of *Lyve1* KO hearts 28 days after MI at P1, a timepoint when regeneration would be anticipated to be complete^15^, revealed aberrant functional parameters. This prompted a reevaluation of the role of LYVE-1 beyond the lymphatic endothelium, drawing on its known expression pattern in tissue-resident macrophages^53^. Intriguingly, macrophage-specific deletion of *Lyve1* provoked an impaired regenerative response after MI, characterized by reduced neovascularization and functional deficits. Mechanistic insight into how *Lyve1* loss of function affects macrophages was provided by scRNA-seq, comparing WT and *Lyve1* KO CD45^+^ cells at P2MI7dpi, which revealed significant reduction in a CCR2^−^ tissue-resident macrophage subpopulation and corresponding elevation in a CCR2^+^ monocyte population, with the loss of CCR2^−^ cells associated with a significantly increased apoptotic score against a panel of established marker genes for programmed cell death. This would be predicted to impair regeneration through exacerbating inflammation and subsequent fibrosis, as per the P7 or adult response. It was previously shown that tissue-resident CCR2^−^ macrophages inhibit inflammatory and pro-fibrotic monocyte recruitment^54^; consequently, the decreased presence of the CCR2^−^ tissue-resident macrophages explains the increased monocyte infiltration, leading to a worse outcome in the KO background. Unbiased differential gene expression analysis confirmed that our identified CCR2^+/−^ macrophage and monocyte clusters aligned with previously reported signatures in terms of effects on angiogenesis, fibrosis and inflammation. These gene expression profiles remained unchanged between WT and KO conditions, strengthening the likelihood that the phenotype observed in the *Lyve1* KO is due to loss of function in CCR2^−^ macrophages, leading to increased inflammatory monocyte recruitment rather than intrinsic changes in molecular phenotype. Notably, the *Lyve1*^+^ macrophage clusters identified in the WT setting were still present in the KO background, albeit without expressing *Lyve1*. This suggests that our observations in the tissue-resident macrophages are *Lyve1* dependent rather than via other mechanisms mediated by macrophages that are lost in the KO background. A putative mechanism as to how LYVE-1 may maintain the CCR2^−^ macrophage population was attributed to establishment and/or maintenance of a hyuralonic-acid-rich glyocaclyx, which is known to protect cells from the exposure of apoptotic membrane cues that trigger phagocytosis^9,38^. This warrants further investigation.

Promoting angiogenesis has been attributed to tissue-resident macrophages previously in the setting of neonatal heart regeneration^21^, and our findings are consistent with this earlier study but also identify an important role for LYVE-1 in orchestrating this response. Elsewhere, bone-marrow-derived LYVE-1^+^ macrophages have a pro-angiogenic role in adipose tissue^55^, but, mechanistically, whether LYVE-1 may directly upregulate angiogenic signaling in tissue-resident macrophages remains unknown. It was reported that LYVE-1 has signaling activity and can function to sequester pro-angiogenic growth factors, including FGF2 and, to a lesser extent, PDGF and VEGF, to induce their internalization and transduce downstream tyrosine kinase receptors and promote endothelial cell proliferation^56^.

Additional insight into roles for LYVE-1, beyond mediating hyuralonic acid adhesion and immune cell trafficking^8^, constitutes an interesting area of follow-up. Further studies to uncover the molecular mechanisms that lead to the differential response across the neonatal regenerative window may provide therapeutic insights into lymphatic-based immunomodulation of the adult infarcted heart.

## Methods

### Mouse strains

The following mouse strains were used for these studies: *Lyve1*^−*/*−^ (ref. ^57^), *hCd68–eGFP*^58^, *CX3CR1-eGFP*^28,59^, *hCd68–CreERT2* (ref. ^36^), *Prox1–tdTomato*^60^, *Vegfr3*^*LacZ/+*^ (ref. ^25^), Gt(ROSA)26Sor^tm9(CAG−tdTomato)Hze^ and *Lyve1*^*flox*^ (Jackson, D. G.; unpublished). Breeding was carried out using only *Cre*^*+*^ males for all Cre strains. Mice were cared for and housed by Oxford University Biomedical Services. Mice were maintained in individually ventilated cages and ventilated racks at 22 °C and 55% humidity. For experiments where WT mice were required, C57BL/6 or CD1 (Charles River Laboratories), when indicated, strains were used. All animal experiments were carried out according to UK Home Office project licenses PPL PC013B246, PDDE89C84 and PP3194787 and were compliant with the UK Animals (Scientific Procedures) Act 1986.

### Timed matings

To generate embryos, female mice were paired with male studs and were checked for vaginal plugs each morning. The day the vaginal plug was observed was designated as embryonic day 0.5.

### Neonatal heart dissection

Neonatal mice were euthanized by cervical dislocation, and the heart was removed. Hearts collected for immunostaining were washed in ice-cold PBS prior to fixation. Hearts for flow cytometry were washed in ice-cold HBSS (Life Technologies) prior to tissue digestion. Hearts for RNA extraction were immediately placed in a cryotube (Nunc; Thermo Fisher Scientific) before submerging in liquid nitrogen.

### Neonatal MI surgery

MI surgery was performed as previously described^61^. At P1, P2 or P7, the litters were removed from the mother and placed in an incubator at 35 °C. General anaesthesia was induced with 4% isoflurane inhalation in oxygen (1 l min^−1^) for 15 seconds. Once unconscious, cardiorespiratory arrest was induced by immersion in ice for 1–2 minutes. The incision site was cleaned with Hibiscrub and a sterile field constructed with drapes. The skin was cut using surgical scissors in a horizontal incision across the left midthorax. Sharp dissecting forceps were used for thoracotomy, and this space was widened using blunt forceps. The heart was manipulated out of the thoracic cavity, and LAD artery ligation was induced by passing and tying a 7.0 prolene suture through the anterior wall of the left ventricle. The sham control procedure involved thoracotomy surgery, heart visualization and suture placement but no ligature. The ribs and skin were then closed in layers with a 7.0 prolene suture. The pup was then warmed under an infrared lamp, which led to gradual return of circulation and breathing. When respiration returned, oxygen was administered via nose cone until regular. The pup was returned to the chamber with other littermates. After surgery was completed on all pups, and before returning the group to the mother, the animals were covered with feces from their cage homogenized in warmed water to mask surgical smells and reduce cannibalization.

### Tamoxifen dosing and administration

Tamoxifen (Sigma-Aldrich) was dissolved in peanut oil containing 10% ethanol by shaking overnight at 37 °C at a concentration of 13.6 mg ml^−1^ before administering to pups at P1 via intraperitoneal injection at a dose of 0.17 mg g^−1^, in line with previous work.

### DNA extraction

Ear biopsies from adult mice and tissue from embryos/neonates were collected for genotyping. The genomic DNA was extracted and amplified using the REDExtract-N-Amp Tissue PCR Kit Protocol (Merck). The tissue was incubated in a mix of 100 μl of Extraction Solution and 25 μl of Tissue Preparation Solution for 10 minutes at room temperature, followed by a 5-minute incubation at 95 °C. Immediately, 100 μl of Neutralization Solution was added, and the mix was vortexed.

### PCR

Extracted DNA was used for genotyping using PCR. The sequence of the primers used is displayed in Supplementary Table 3. The following reagents were used to set up the PCR reactions: 10 μl of REDExtraction-N-Amp PCR Reaction Mix, 0.5 μM of each primer, 4 μl of tissue extract and Milli-Q water until total reaction volume of 20 μl. Thermal cycling was carried out in a Veriti 96-well thermal cycler (Applied Biosystems). A positive control sample of known genotype was included for each genotype being tested.

### Agarose gel electrophoresis

After PCR amplification, the DNA was separated on 1.5% agarose gel. Then, 1.5 g of agarose (Sigma-Aldrich) was dissolved in 100 ml of TBE buffer by heating the mix in a microwave. For ultraviolet visualization of DNA, 5 μl of GelRed (VWR) was added to the agarose gel before it set in moulds. An electric potential of 160 V was passed through the gel for 35 minutes to allow separation of bands to distinguish between genotypes. Gels were visualized in an ultraviolet box.

### RNA extraction from tissue

RNA was isolated from snap-frozen tissue samples using TRIzol reagent (Thermo Fisher Scientific). Tissue was homogenized in 750 μl of TRIzol solution using a manual homogenizer and a 21-gauge sterile needle (Becton Dickinson). After being incubated for 5 minutes at room temperature, 200 μl of chloroform was added to the samples. Samples were then mixed and incubated for 15 minutes at room temperature. After incubation, samples were centrifuged at 11,000*g* for 15 minutes at 4 °C. The top aqueous layer was transferred into a 1.5-ml tube, and the organic layer was discarded. Next, 500 μl of isopropanol was added to precipitate RNA and incubated at 4 °C overnight. The samples were then centrifuged at 11,000*g* for 10 minutes to produce an RNA-containing pellet. The pellet was washed with 1 ml of 75% ethanol before spinning at 9,000*g* for 5 minutes at 4 °C. The pellet was air dried for 10 minutes and resuspended in diethyl pyrocarbonate (DEPC)-treated water. A NanoDrop 2000 (Thermo Fisher Scientific) was used to measure RNA quality and concentration.

### cDNA synthesis

cDNA was synthesized from extracted RNA for use in real-time quantitative PCR (qRT–PCR). Reactions were prepared in RNase-free 0.6-ml tubes (Thermo Fisher Scientific) using the following reagents: 1 μg of RNA made up to a volume of 8.5 μl with DEPC water, 0.5 μl of random primers (20 μg ml^−1^; Promega), 1 μl of dNTPs (from 10 mM; GE Healthcare), 2 μl of MgCl_2_ (25 mM; Thermo Fisher Scientific), 2 μl of dithiothreitol (0.1 M; Life Technologies), 1 μl of RNasin plus RNase inhibitor (Promega), 4 μl of 5× FS Buffer (Life Technologies) and 1 μl of SuperScript III Reverse Transcriptase (Life Technologies). A Veriti 96-well thermal cycler (Applied Biosystems) was used to run the reaction at 25 °C for 10 minutes, 42 °C for 50 minutes and 70 °C for 15 minutes, before cooling to 4 °C. After cDNA synthesis, samples were diluted to 4 ng μl^−1^ in DEPC-treated water and stored at 4 °C prior (short-term) to use in qRT–PCR experiments.

### qRT–PCR

Relative mRNA expression levels from genes of interest were quantified using qRT–PCR. Primer sequences are displayed in Supplementary Table 4. MicroAmp Fast Optical 96-well 0.1-ml reaction plates (Thermo Fisher Scientific) were used to set up reactions, which were composed of the following reagents: 8 ng of cDNA, 13 μl of Fast SYBR Green Master Mix (Thermo Fisher Scientific), 6.5 μl of DEPC-treated water and 0.5 μM of each primer. All samples were run in triplicate, and a no-cDNA negative control well was included for each gene analyzed. Reactions were run on a ViiA7 Real-Time PCR System (Thermo Fisher Scientific) with thermal cycles of 95 °C for 15 minutes, 40 cycles of 95 °C for 15 seconds and 60 °C for 1 minute. Melt curves were included to confirm that no unspecific amplification products, such as primer dimers, were produced with each primer set used.

Cycle threshold values were obtained and exported to Microsoft Excel for analysis.

### Histological analyses and staining

#### Whole-mount X-Gal staining

After extraction, hearts were washed in ice-cold PBS and then fixed in X-Gal fixation solution (Supplementary Table 5) for 6 hours at room temperature. Then, hearts were washed in X-Gal buffer and incubated in X-Gal staining solution (Supplementary Table 5) overnight at 37 °C. The next day, fresh X-Gal staining solution was added and left overnight at 37 °C. Once staining had developed, hearts were washed in X-Gal buffer and post-fixed in 4% paraformaldehyde (PFA) for 30 minutes. Finally, hearts were washed in PBS and imaged using a Zeiss stereomicroscope. Analysis of vessels and branching calculations were performed using AngioTool software.

#### Whole-mount tissue clearing

Whole hearts were cleared using an iDISCO (immunolabeling-enabled three-dimensional imaging of solvent-cleared organs) protocol adapted from previous work^62^. Hearts were dehydrated by methanol/water series (20%, 40%, 60%, 80%, 100%, each 1 hour) and pretreated with 66% dichloromethane, 33% methanol at room temperature overnight. Samples were washed in methanol twice before bleaching in chilled fresh 5% H_2_O_2_ in methanol, shaking overnight. Hearts were then rehydrated by methanol/water series (80%, 60%, 40%, 20%, PBS, 1 hour each). Pretreated samples were washed in 0.2% Triton X-100 PBS before incubation in permeabilization solution (0.2% Triton X-100, 0.3 M glycine, 20% DMSO, PBS) for 2 days, shaking at 37 °C. Hearts were then moved to blocking solution (0.2% Triton X-100, 6% donkey serum, 10% DMSO, PBS) for 2 days, shaking at 37 °C. Hearts were then incubated in primary antibody solution (1:300 in 0.2% Tween 20, 10 mg ml^−1^ heparin, 3% donkey serum, 5% DMSO, PBS) for 7 days, shaking at 37 °C. Samples were then washed 5 × 1 hour in 0.2% Tween 20, 10 mg ml^−1^ heparin PBS, shaking at room temperature. Hearts were subsequently incubated in secondary antibody (1:300 in 0.2% Tween 20, 10 mg ml^−1^ heparin, 3% donkey serum, PBS) for 7 days, shaking at 37 °C, before being washed 5 × 1 hour in 0.2% Tween 20, 10 mg ml^−1^ heparin PBS. Hearts were then dehydrated in methanol/water series and incubated in 66% dichloromethane, 33% methanol for 3 hours. Samples were then moved to 100% dichloromethane for 2 × 15 minutes before incubation in dibenzyl ether for 2 days to clear the tissue. Samples were refractive index matched by moving to ethyl cinnamate overnight. Whole-mount samples were imaged in ethyl cinnamate using an LaVision UltraMicroscope II light-sheet microscope.

#### Picrosirius red staining

Staining was carried out using Picrosirius staining kits (Abcam) according to the manufacturer’s protocol. Sections were hydrated in distilled water and immersed in Picrosirius red solution for 1 hour before being rinsed in two changes of 1% acetic acid followed by two changes of absolute ethanol. Sections were then cleared in Histo-Clear II (National Diagnostics) and mounted with DPX (Sigma-Aldrich). Images of sections were obtained using a Nikon Eclipse light microscope.

#### Paraffin embedding

Hearts for histological and immunofluorescent staining were embedded in paraffin and mounted using standard methods. In brief, fixed hearts were dehydrated through an ethanol gradient (50% 1 hour, 70% 1 hour, 90% 1 hour, 100% overnight) and cleared with Histo-Clear II for 1 hour. Samples were then embedded first in a 1:1 mixture of Histo-Clear II and Paraplast Plus (Sigma-Aldrich) at 60 °C overnight and then in 100% Paraplast Plus at 60 °C overnight before returning to room temperature and setting. Then, 10-μm sections were cut and mounted onto Superfrost slides. Prior to staining, sections were deparaffinized in Histo-Clear II and a reversed ethanol gradient (100% 3 minutes, 90% 3 min, 70% 3 minutes, 50% 3 minutes) before transfer to PBS.

#### Cryosectioning

Whole neonatal hearts were fixed in 4% PFA overnight at 4 °C. After fixation, samples were washed three times for 10 minutes in PBS and then transferred to 30% sucrose and PBS overnight at 4 °C. Then, the samples were equilibrated in a 1:1 solution of Optimal Cutting Temperature (OCT) and 30% sucrose for 1 hour in 4 °C. After equilibration, the hearts were embedded in 100% OCT and frozen at −80 °C. Next, 20–25-μm slices were cut using a cryostat and transferred onto Superfrost Plus slides (VMR). Slides were dried on a slide dryer for 15 minutes before being rinsed with PBS.

#### Immunostaining

Sections underwent permeabilization with 0.5% Triton X-100 (Sigma-Aldrich) for 10 minutes, followed by two rinses in PBS for 5 minutes. Then, sections were blocked in blocking solution, composed of 10% serum, 4% BSA and 0.2% Triton X-100, for 1 hour. Blocking was followed by a 4 °C overnight incubation in primary antibody, which was diluted in blocking solution. A list of primary antibodies and the dilutions used is included in Supplementary Table 6. After primary antibody incubation, sections were washed several times with 0.1% Triton X-100 and incubated with Alexa Fluor-conjugated secondary antibodies for 1 hour at room temperature in the dark. All secondary antibodies were diluted in PBS; a list of the secondary antibodies used is included in Supplementary Table 7. After incubation in secondary antibody, the slides were washed several times in 0.1% Triton X-100, with DAPI included in the final 15-minute wash. A small amount of 50:50 glycerol:PBS was then added to the slides, and a 22 × 50-mm coverslip (Fisherbrand) was placed on top and sealed with nail varnish. Whole hearts were stained using the same protocol but were permeabilized for 1 hour, blocked overnight, stained with primary antibodies for 48 hours and stained for secondary antibodies overnight. Immunofluorescent staining was imaged using a Zeiss LSM780, a Zeiss LSM880, a Zeiss LSM980 or a Leica confocal microscope. A Zeiss Z.1 light-sheet microscope was used to obtain whole-mount images. *z*-stack and tiling functions were used when required. Images were processed using Imaris, Arivis Vision4D and ImageJ software^63^.

#### Flow cytometry: neonatal cardiac cell isolation

Hearts were isolated, the atria were removed and the ventricles were bisected. Blood was removed from the ventricles, which were minced using a sterile scalpel (Swann-Morton) before being added to a 15-ml tube containing 5 ml of HBSS (Life Technologies) and 500 U ml^−1^ Collagenase II (Worthington). The tubes were placed in an orbital incubator at 37 °C, moving at 190 r.p.m. Plastic transfer pipettes were used to disrupt the suspension every 10 minutes during the 30-minute digestion within the orbital incubator. Once digested, the tissue suspension was passed through a 40-μm cell strainer attached to a 50-ml tube. The tubes were then centrifuged at 350*g* for 10 minutes at 4 °C, and the supernatant was discarded, leaving a pellet. The pellet was resuspended in 5 ml of 1× red blood cell (RBC) lysis buffer (BioLegend) and was left at room temperature for 10 minutes. The tubes were then centrifuged again at 350*g* for 5 minutes at 4 °C, and the supernatant was discarded, leaving a pellet. The cell pellet was resuspended in 5 ml of 2% FBS/PBS. The tubes were centrifuged again at 350*g* for 5 minutes at 4 °C, and the supernatant was discarded, leaving a pellet. The pellet containing the cardiac cells was resuspended in 200 μl of 2% FBS/PBS.

For scRNA-seq, the pellet was resuspended in 200 μl of 2% FBS/PBS and transferred to a Falcon 5-ml round-bottom polystyrene test tube, with a 35-μm nylon mesh Cell Strainer Snap Cap prior to fluorescence-activated cell sorting (FACS) of live cells using 7-AAD.

#### Adoptive transfer studies

Monocytes were isolated from the spleen of *hCd68–eGFP*^*+*^ adult mice. After cervical dislocation, the spleen was dissected and disrupted with the blunt end of a sterile syringe. The disrupted spleen was washed through a 70-μm cell filter into RBC lysis buffer and incubated at room temperature for 10 minutes. Cells were then spun down (400*g* for 5 minutes), and RBC lysis buffer was removed by aspiration. Monocytes were purified from the cell pellet using an EasySep Mouse Monocyte Enrichment Kit according to the manufacturer’s instructions. Prior to surgery, cells were counted and resuspended in PBS to give a final concentration of 50 × 10^3^ per 5 μl for injection. Each recipient received 1 ×5-μl injection of cells by intracardiac injection (using a 30-gauge insulin syringe) at the time of MI surgery.

#### MRI

Neonatal cardiac cine MRI was performed after MI at day 28 as previously described^14^ using a 7T preclinical MR system (Varian) using a ^1^H four-channel phased array surface receive coil (RAPID Biomedical). In brief, mice were anesthetized with 2% isoflurane in O_2_ and positioned prone in a custom animal handling system with homeothermic control. Prospectively gated proton cardiac images were acquired with a partial Fourier accelerated spoiled gradient echo cine sequence (TR 5.9 ms, TE 2.2 ms, 30 kHz bandwidth, 30° flip angle, approximately 20–30 frames; 3–4 averages) in order to acquire two-chamber and four-chamber long-axis views and a stack of contiguous 1-mm-thick true short-axis images to cover the entire left ventricle (128 × 128 matrix; 25.6 mm^2^ field of view; 0.2-mm resolution in-plane).

#### MRI data analysis

MRI data were processed manually by image analysis with ImageJ. All analysis was performed blinded so as the intact/surgery and WT/mutant groups were unknown at the time of image processing. Measurements were calculated as previously described^14^. End diastolic and end systolic volumes were measured for each slice and summed over the whole heart. StV was calculated by subtracting the end systolic volume from the end diastolic volume. Ejection fraction was calculated by dividing the end diastolic volume by the StV. EDM was calculated as the left ventricular end diastolic volume multiplied by the myocardial specific gravity (1.05 g cm^−^^3^); ESM was also calculated in this way using the left ventricular end systolic volume. Cardiac output was calculated as StV multiplied by heart rate. The relative infarct size was calculated from the average of the endocardial and epicardial circumferential lengths of the thinned, akinetic region of all slices, measured at diastole and expressed as a percentage of the total myocardial surface.

### scRNA-seq and analysis

#### 10x sequencing

All the 10x scRNA library preparation and sequencing was carried out at the Oxford Genomics Centre, part of the Wellcome Trust Centre for Human Genetics.

#### Bioinformatics analysis

The raw scRNA-seq data were demultiplexed by Adam Braithwaite (Radcliffe Department of Medicine) using cellranger mkfastq from the Cell Ranger software suite to generate FASTQ files. Demultiplexed FASTQ files were aligned to the mouse mm10/GRCm38 reference transcriptome, and the gene expression matrix was counted using the cellranger ‘count’ program. Downstream bioinformatics analysis was performed in R using the Seurat package (version 4)^64^. A cutoff was applied to filter out low-quality cells based on the number of genes detected (<500), the number of unique molecular identifiers detected (<250) and the mitochondrial gene content (>30%) per each cell. Data from all samples were combined, scaled by regressing out S and G2 cell cycle phases and integrated using the Seurat package. Dimensional reduction of the data was performed by the RunUMAP function implemented in the Seurat package. Unsupervised cluster identification was performed using the FindClusters function, and cell clusters were further annotated based on expression of known marker genes^32^. Identification of enriched genes in cell clusters and single-cell differential expression analysis were performed using the Seurat functions FindMarkers and FindAllMarkers, using gene expression detected in more than 25% of cells for at least one of the populations being compared, with an absolute log_2_(fold change) > 0.25 as cutoff threshold.

To genetically demultiplex pooled scRNA-seq samples (*n* = 5 hearts), we remapped 10x scRNA-seq reads using minimap^65^, called variants using freebayes and counted alleles in each cell using VarTrix, all as part of the souporcell pipeline^66^. Based on the VarTrix output, cells were assigned to donors using Vireo5 (ref. ^37^). We excluded doublet and unassigned cells from further analysis and performed differential abundance testing of cell neighborhoods between WT and *Lyve1* KO conditions using miloR^67^, building the *k*-nearest neighbor (KNN) graph with *k* = 30. This tool represents scRNA-seq data as a KNN graph consisting of partially overlapping cell neighborhoods that contain an index cell and its nearest neighbor cells. Neighborhoods are annotated to the cell type that they contain most frequently, and a representative sample of neighborhoods was tested for differential abundance across conditions.

Apoptosis markers were retrieved from the Molecular Signatures Database (HALLMARK APOPTOSIS), and a cumulative expression score was calculated using Seurat’s AddModuleScore() function. Markers used to analyze G1/S and G2/M cell cycle phase transitions^68^ are included in the Seurat R package. The CellCycleScoring() function was used to calculate cumulative expression scores of cell cycle markers. Statistical differences in the distribution of single-cell gene expression between conditions were evaluated using the Wilcoxon rank-sum test, and *P* values were Bonferroni corrected to account for multiple comparisons.

#### Statistical analysis

All statistical analyses were performed using GraphPad Prism 8 software. Statistical tests are described in the figure legends. In all cases, *P* values less than or equal to 0.05 were deemed significant.

### Reporting summary

Further information on research design is available in the Nature Portfolio Reporting Summary linked to this article.

## Supplementary information

Reporting Summary

Supplementary Tables 1–7 Supplementary Table 1: Number of cells and differentially expressed genes (DEGs) for scRNA-seq analysis of P1 and P7 hearts, uninjured and 1 day and 7 days after infarct. Supplementary Table 2: Macrophage numbers for scRNA-seq analysis of WT versus *Lyve1* KO at P2MI7dpi. Supplementary Table 3: PCR primers for genotyping. Supplementary Table 4: Primers for qRT–PCR. Supplementary Table 5: Histology solutions. Supplementary Table 6: List of primary antibodies. Supplementary Table 7: List of secondary antibodies.

## Source data

Source Data Fig. 1–5,7,8 and Source Data Extended Data Fig. 1,3,4,6,7–10 Statistical source data.

## Data Availability

The scRNA-seq datasets generated in this study are deposited in the Gene Expression Omnibus with accession number GSE301633.

## References

[1] Klotz, L. et al. Cardiac lymphatics are heterogeneous in origin and respond to injury. *Nature* **522**, 62–67 (2015). 25992544 10.1038/nature14483 PMC4458138

[2] Henri, O. et al. Selective stimulation of cardiac lymphangiogenesis reduces myocardial edema and fibrosis leading to improved cardiac function following myocardial infarction. *Circulation* **133**, 1484–1497 (2016). 26933083 10.1161/CIRCULATIONAHA.115.020143

[3] Vieira, J. M. et al. The cardiac lymphatic system stimulates resolution of inflammation following myocardial infarction. *J. Clin. Invest.* **128**, 3402–3412 (2018). 29985167 10.1172/JCI97192 PMC6063482

[4] Houssari, M. et al. Lymphatic and immune cell cross-talk regulates cardiac recovery after experimental myocardial infarction. *Arterioscler. Thromb. Vasc. Biol.* **40**, 1722–1737 (2020). 10.1161/ATVBAHA.120.314370 PMC7310303 32404007

[5] Rurik, J. G., Aghajanian, H. & Epstein, J. A. Immune cells and immunotherapy for cardiac injury and repair. *Circ. Res.* **128**, 1766–1779 (2021). 34043424 10.1161/CIRCRESAHA.121.318005 PMC8171813

[6] Adamo, L., Rocha-Resende, C., Prabhu, S. D. & Mann, D. L. Reappraising the role of inflammation in heart failure. *Nat. Rev. Cardiol.* **17**, 269–285 (2020). 31969688 10.1038/s41569-019-0315-x

[7] Jackson, D. G. Hyaluronan in the lymphatics: the key role of the hyaluronan receptor LYVE-1 in leucocyte trafficking. *Matrix Biol.* **78-79**, 219–235 (2019). 29425695 10.1016/j.matbio.2018.02.001

[8] Johnson, L. A. et al. Dendritic cells enter lymph vessels by hyaluronan-mediated docking to the endothelial receptor LYVE-1. *Nat. Immunol.* **18**, 762–770 (2017). 28504698 10.1038/ni.3750

[9] Johnson, L. A., Banerji, S., Lagerholm, B. C. & Jackson, D. G. Dendritic cell entry to lymphatic capillaries is orchestrated by CD44 and the hyaluronan glycocalyx. *Life Sci. Alliance* **4**, e202000908 (2021). 10.26508/lsa.202000908 PMC8008951 33687996

[10] Johnson, L. A. & Jackson, D. G. Hyaluronan and its receptors: key mediators of immune cell entry and trafficking in the lymphatic system. *Cells* **10**, 2061 (2021). 34440831 10.3390/cells10082061 PMC8393520

[11] Lawrance, W., Banerji, S., Day, A. J., Bhattacharjee, S. & Jackson, D. G. Binding of hyaluronan to the native lymphatic vessel endothelial receptor LYVE-1 is critically dependent on receptor clustering and hyaluronan organization. *J. Biol. Chem.* **291**, 8014–8030 (2016). 26823460 10.1074/jbc.M115.708305 PMC4825007

[12] Bano, F. et al. Structure and unusual binding mechanism of the hyaluronan receptor LYVE-1 mediating leucocyte entry to lymphatics. *Nat. Commun.* **16**, 2754 (2025). 40113779 10.1038/s41467-025-57866-8 PMC11926218

[13] Lam, N. T. & Sadek, H. A. Neonatal heart regeneration: comprehensive literature review. *Circulation* **138**, 412–423 (2018). 30571359 10.1161/CIRCULATIONAHA.118.033648 PMC6673675

[14] Gunadasa-Rohling, M. et al. Magnetic resonance imaging of the regenerating neonatal mouse heart. *Circulation* **138**, 2439–2441 (2018). 30571586 10.1161/CIRCULATIONAHA.118.036086

[15] Porrello, E. R. et al. Regulation of neonatal and adult mammalian heart regeneration by the miR-15 family. *Proc. Natl Acad. Sci. USA* **110**, 187–192 (2013). 23248315 10.1073/pnas.1208863110 PMC3538265

[16] Saker, D. M., Walsh-Sukys, M., Spector, M. & Zahka, K. G. Cardiac recovery and survival after neonatal myocardial infarction. *Pediatr. Cardiol.* **18**, 139–142 (1997). 9049129 10.1007/s002469900133

[17] Haubner, B. J. et al. Functional recovery of a human neonatal heart after severe myocardial infarction. *Circ. Res.* **118**, 216–221 (2016). 26659640 10.1161/CIRCRESAHA.115.307017

[18] Lavine, K. J. et al. Distinct macrophage lineages contribute to disparate patterns of cardiac recovery and remodeling in the neonatal and adult heart. *Proc. Natl Acad. Sci. USA* **111**, 16029–16034 (2014). 25349429 10.1073/pnas.1406508111 PMC4234568

[19] Wang, Z. et al. Mechanistic basis of neonatal heart regeneration revealed by transcriptome and histone modification profiling. *Proc. Natl Acad. Sci. USA* **116**, 18455–18465 (2019). 31451669 10.1073/pnas.1905824116 PMC6744882

[20] Molawi, K. et al. Progressive replacement of embryo-derived cardiac macrophages with age. *J. Exp. Med.* **211**, 2151–2158 (2014). 25245760 10.1084/jem.20140639 PMC4203946

[21] Aurora, A. B. et al. Macrophages are required for neonatal heart regeneration. *J. Clin. Invest.* **124**, 1382–1392 (2014). 24569380 10.1172/JCI72181 PMC3938260

[22] Du Cheyne, C., Tay, H. & De Spiegelaere, W. The complex TIE between macrophages and angiogenesis. *Anat. Histol. Embryol.* **49**, 585–596 (2020). 31774212 10.1111/ahe.12518

[23] Liu, X. et al. Lymphoangiocrine signals promote cardiac growth and repair. *Nature* **588**, 705–711 (2020). 33299187 10.1038/s41586-020-2998-x PMC7770123

[24] Dumont, D. J. et al. Cardiovascular failure in mouse embryos deficient in VEGF receptor-3. *Science* **282**, 946–949 (1998). 9794766 10.1126/science.282.5390.946

[25] Gancz, D. et al. Distinct origins and molecular mechanisms contribute to lymphatic formation during cardiac growth and regeneration. *eLife* **8**, e44153 (2019). 10.7554/eLife.44153 PMC6881115 31702554

[26] Russo, E. et al. Intralymphatic CCL21 promotes tissue egress of dendritic cells through afferent lymphatic vessels. *Cell Rep.* **14**, 1723–1734 (2016). 26876174 10.1016/j.celrep.2016.01.048

[27] Weber, M. et al. Interstitial dendritic cell guidance by haptotactic chemokine gradients. *Science* **339**, 328–332 (2013). 23329049 10.1126/science.1228456

[28] Zaman, R. & Epelman, S. Resident cardiac macrophages: heterogeneity and function in health and disease. *Immunity* **55**, 1549–1563 (2022). 36103852 10.1016/j.immuni.2022.08.009

[29] Baluk, P. et al. Functionally specialized junctions between endothelial cells of lymphatic vessels. *J. Exp. Med.* **204**, 2349–2362 (2007). 17846148 10.1084/jem.20062596 PMC2118470

[30] Yao, L. C., Baluk, P., Srinivasan, R. S., Oliver, G. & McDonald, D. M. Plasticity of button-like junctions in the endothelium of airway lymphatics in development and inflammation. *Am. J. Pathol.* **180**, 2561–2575 (2012). 22538088 10.1016/j.ajpath.2012.02.019 PMC3378913

[31] Baluk, P. & McDonald, D. M. Buttons and zippers: endothelial junctions in lymphatic vessels. *Cold Spring Harb. Perspect. Med.* **12**, a041178 (2022). 10.1101/cshperspect.a041178 PMC9643678 35534209

[32] Wang, Z. et al. Cell-type-specific gene regulatory networks underlying murine neonatal heart regeneration at single-cell resolution. *Cell Rep.* **33**, 108472 (2020). 33296652 10.1016/j.celrep.2020.108472 PMC7774872

[33] Farbehi, N. et al. Single-cell expression profiling reveals dynamic flux of cardiac stromal, vascular and immune cells in health and injury. *eLife* **8**, e43882 (2019). 10.7554/eLife.43882 PMC6459677 30912746

[34] Zhang, Y. et al. Dedifferentiation and proliferation of mammalian cardiomyocytes. *PLoS ONE* **5**, e12559 (2010). 20838637 10.1371/journal.pone.0012559 PMC2933247

[35] Gale, N. W. et al. Angiopoietin-2 is required for postnatal angiogenesis and lymphatic patterning, and only the latter role is rescued by Angiopoietin-1. *Dev. Cell* **3**, 411–423 (2002). 12361603 10.1016/s1534-5807(02)00217-4

[36] Rumianek, A. N., Davies, B., Channon, K. M., Greaves, D. R. & Purvis, G. S. D. A human CD68 promoter-driven inducible Cre-recombinase mouse line allows specific targeting of tissue resident macrophages. *Front. Immunol.* **13**, 918636 (2022). 35874787 10.3389/fimmu.2022.918636 PMC9298978

[37] Huang, Y., McCarthy, D. J. & Stegle, O. Vireo: Bayesian demultiplexing of pooled single-cell RNA-seq data without genotype reference. *Genome Biol.* **20**, 273 (2019). 31836005 10.1186/s13059-019-1865-2 PMC6909514

[38] Le, T. et al. Redistribution of the glycocalyx exposes phagocytic determinants on apoptotic cells. *Dev. Cell* **59**, 853–868 (2024). 38359833 10.1016/j.devcel.2024.01.020

[39] Gancz, D., Perlmoter, G. & Yaniv, K. Formation and growth of cardiac lymphatics during embryonic development, heart regeneration, and disease. *Cold Spring Harb. Perspect. Biol.* **12**, a037176 (2020). 10.1101/cshperspect.a037176 PMC7263081 31818858

[40] Stone, O. A. & Stainier, D. Y. R. Paraxial mesoderm is the major source of lymphatic endothelium. *Dev. Cell* **50**, 247–255 (2019). 31130354 10.1016/j.devcel.2019.04.034 PMC6658618

[41] Lioux, G. et al. A second heart field-derived vasculogenic niche contributes to cardiac lymphatics. *Dev. Cell* **52**, 350–363 (2020). 31928974 10.1016/j.devcel.2019.12.006 PMC7374559

[42] Stevenson Keller 4th, T. C. et al. Genetic blockade of lymphangiogenesis does not impair cardiac function after myocardial infarction. *J. Clin. Invest.* **131**, e147070 (2021). 10.1172/JCI147070 PMC8516448 34403369

[43] Poss, K. D., Wilson, L. G. & Keating, M. T. Heart regeneration in zebrafish. *Science* **298**, 2188–2190 (2002). 12481136 10.1126/science.1077857

[44] Gonzalez-Rosa, J. M., Martin, V., Peralta, M., Torres, M. & Mercader, N. Extensive scar formation and regression during heart regeneration after cryoinjury in zebrafish. *Development* **138**, 1663–1674 (2011). 21429987 10.1242/dev.060897

[45] Stockdale, W. T. et al. Heart regeneration in the Mexican cavefish. *Cell Rep.* **25**, 1997–2007 (2018). 30462998 10.1016/j.celrep.2018.10.072 PMC6280125

[46] Porrello, E. R. et al. Transient regenerative potential of the neonatal mouse heart. *Science* **331**, 1078–1080 (2011). 21350179 10.1126/science.1200708 PMC3099478

[47] Harrison, M. R. et al. Late developing cardiac lymphatic vasculature supports adult zebrafish heart function and regeneration. *eLife* **8**, e42762 (2019). 10.7554/eLife.42762 PMC6881116 31702553

[48] Vivien, C. J. et al. Vegfc/d-dependent regulation of the lymphatic vasculature during cardiac regeneration is influenced by injury context. *NPJ Regen. Med.* **4**, 18 (2019). 31452940 10.1038/s41536-019-0079-2 PMC6706389

[49] Hilgendorf, I. et al. Ly-6C^high^ monocytes depend on Nr4a1 to balance both inflammatory and reparative phases in the infarcted myocardium. *Circ. Res.* **114**, 1611–1622 (2014). 24625784 10.1161/CIRCRESAHA.114.303204 PMC4017349

[50] Schmid-Schonbein, G. W. The second valve system in lymphatics. *Lymphat. Res. Biol.* **1**, 25–29; discussion 29–31 (2003). 10.1089/15396850360495664 15624318

[51] Pflicke, H. & Sixt, M. Preformed portals facilitate dendritic cell entry into afferent lymphatic vessels. *J. Exp. Med.* **206**, 2925–2935 (2009). 19995949 10.1084/jem.20091739 PMC2806476

[52] Travisano, S. I. et al. Single-nuclei multiomic analyses identify human cardiac lymphatic endothelial cells associated with coronary arteries in the epicardium. *Cell Rep.* **42**, 113106 (2023). 37676760 10.1016/j.celrep.2023.113106

[53] Cahill, T. J. et al. Tissue-resident macrophages regulate lymphatic vessel growth and patterning in the developing heart. *Development* **148**, dev194563 (2021). 10.1242/dev.194563 PMC7875498 33462113

[54] Bajpai, G. et al. Tissue resident CCR2^−^ and CCR2^+^ cardiac macrophages differentially orchestrate monocyte recruitment and fate specification following myocardial injury. *Circ. Res.* **124**, 263–278 (2019). 30582448 10.1161/CIRCRESAHA.118.314028 PMC6626616

[55] Cho, C. H. et al. Angiogenic role of LYVE-1-positive macrophages in adipose tissue. *Circ. Res.* **100**, e47–e57 (2007). 17272806 10.1161/01.RES.0000259564.92792.93

[56] Platonova, N. et al. Evidence for the interaction of fibroblast growth factor-2 with the lymphatic endothelial cell marker LYVE-1. *Blood* **121**, 1229–1237 (2013). 23264596 10.1182/blood-2012-08-450502

[57] Gale, N. W. et al. Normal lymphatic development and function in mice deficient for the lymphatic hyaluronan receptor LYVE-1. *Mol. Cell. Biol.* **27**, 595–604 (2007). 17101772 10.1128/MCB.01503-06 PMC1800809

[58] Iqbal, A. J. et al. Human CD68 promoter GFP transgenic mice allow analysis of monocyte to macrophage differentiation in vivo. *Blood* **124**, e33–e44 (2014). 25030063 10.1182/blood-2014-04-568691 PMC4192756

[59] Jung, S. et al. Analysis of fractalkine receptor CX_3_CR1 function by targeted deletion and green fluorescent protein reporter gene insertion. *Mol. Cell. Biol.* **20**, 4106–4114 (2000). 10805752 10.1128/mcb.20.11.4106-4114.2000 PMC85780

[60] Truman, L. A. et al. ProxTom lymphatic vessel reporter mice reveal Prox1 expression in the adrenal medulla, megakaryocytes, and platelets. *Am. J. Pathol.* **180**, 1715–1725 (2012). 22310467 10.1016/j.ajpath.2011.12.026 PMC3349900

[61] De Villiers, C. & Riley, P. R. A refined protocol for coronary artery ligation in the neonatal mouse. *Curr. Protoc.* **1**, e66 (2021). 33617028 10.1002/cpz1.66

[62] Renier, N. et al. iDISCO: a simple, rapid method to immunolabel large tissue samples for volume imaging. *Cell* **159**, 896–910 (2014). 25417164 10.1016/j.cell.2014.10.010

[63] Rueden, C. T. et al. ImageJ2: ImageJ for the next generation of scientific image data. *BMC Bioinformatics* **18**, 529 (2017). 29187165 10.1186/s12859-017-1934-z PMC5708080

[64] Stuart, T. et al. Comprehensive integration of single-cell data. *Cell* **177**, 1888–1902 (2019). 31178118 10.1016/j.cell.2019.05.031 PMC6687398

[65] Li, H. Minimap2: pairwise alignment for nucleotide sequences. *Bioinformatics* **34**, 3094–3100 (2018). 29750242 10.1093/bioinformatics/bty191 PMC6137996

[66] Heaton, H. et al. Souporcell: robust clustering of single-cell RNA-seq data by genotype without reference genotypes. *Nat. Methods* **17**, 615–620 (2020). 32366989 10.1038/s41592-020-0820-1 PMC7617080

[67] Dann, E., Henderson, N. C., Teichmann, S. A., Morgan, M. D. & Marioni, J. C. Differential abundance testing on single-cell data using *k*-nearest neighbor graphs. *Nat. Biotechnol.* **40**, 245–253 (2022). 34594043 10.1038/s41587-021-01033-z PMC7617075

[68] Tirosh, I. et al. Single-cell RNA-seq supports a developmental hierarchy in human oligodendroglioma. *Nature* **539**, 309–313 (2016). 27806376 10.1038/nature20123 PMC5465819

