# Supplementary material for: Cardiac lymphatics retain LYVE-1-dependent macrophages during neonatal mouse heart regeneration
Source: Nat Cardiovasc Res. 2025 Sep 17;4(10):1258–76. doi: 10.1038/s44161-025-00711-4 (PMC12520984; doi:10.1038/s44161-025-00711-4)
Supplement: Supplementary file 1 — Reporting Summary [file 44161_2025_711_MOESM1_ESM.pdf]

nature portfolio

Corresponding author(s): Professor Paul R. Riley
Dr Joaquim M. Vieira

Last updated by author(s): Jul 27, 2025

Reporting Summary

Nature Portfolio wishes to improve the reproducibility of the work that we publish. This form provides structure for consistency and transparency in reporting. For further information on Nature Portfolio policies, see our Editorial Policies and the Editorial Policy Checklist.

Statistics

For all statistical analyses, confirm that the following items are present in the figure legend, table legend, main text, or Methods section.

n/a	Confirmed	
☐	☒	The exact sample size (*n*) for each experimental group/condition, given as a discrete number and unit of measurement
☐	☒	A statement on whether measurements were taken from distinct samples or whether the same sample was measured repeatedly
☐	☒	The statistical test(s) used AND whether they are one- or two-sided<br>*Only common tests should be described solely by name; describe more complex techniques in the Methods section.*
☒	☐	A description of all covariates tested
☐	☒	A description of any assumptions or corrections, such as tests of normality and adjustment for multiple comparisons
☐	☒	A full description of the statistical parameters including central tendency (e.g. means) or other basic estimates (e.g. regression coefficient) AND variation (e.g. standard deviation) or associated estimates of uncertainty (e.g. confidence intervals)
☐	☒	For null hypothesis testing, the test statistic (e.g. *F*, *t*, *r*) with confidence intervals, effect sizes, degrees of freedom and *P* value noted<br>*Give P values as exact values whenever suitable.*
☒	☐	For Bayesian analysis, information on the choice of priors and Markov chain Monte Carlo settings
☒	☐	For hierarchical and complex designs, identification of the appropriate level for tests and full reporting of outcomes
☒	☐	Estimates of effect sizes (e.g. Cohen's *d*, Pearson's *r*), indicating how they were calculated

Our web collection on statistics for biologists contains articles on many of the points above.

Software and code

Policy information about availability of computer code

Data collection	Zeiss ZEN Blue microscopy software (v3.12) was used to capture confocal microscopy images
Data analysis	ImageJ (v1.54) was used to process and quantify microscopy images and MRI data, Adobe Photoshop (v25.12.3) was used to produce figures, Imaris (v10.2) was used to process and quantify microscopy images, GraphPad Prism (v10.0.3) was used to produce graphs and perform statistical analysis, AngioTool (PMID: 22110636) was used to quantify lymphatic vessels, Zeiss Arivis Vision4D (v4.1.2) was used to process whole mount heart z-stacks, Seurat R package (v5.3.0) was used to process scRNA-Seq data, 10x Genomics Cell Ranger suite (v9.0.1), VarTrix (v1.1.14), Souporcell pipline (v2.5), Vireo (PMID: 31836005), and miloR (v3.20) were used to demultiplex scRNA-Seq data.

For manuscripts utilizing custom algorithms or software that are central to the research but not yet described in published literature, software must be made available to editors and reviewers. We strongly encourage code deposition in a community repository (e.g. GitHub). See the Nature Portfolio guidelines for submitting code & software for further information.

Data

Policy information about availability of data

All manuscripts must include a data availability statement. This statement should provide the following information, where applicable:

- Accession codes, unique identifiers, or web links for publicly available datasets
- A description of any restrictions on data availability
- For clinical datasets or third party data, please ensure that the statement adheres to our policy

The single-cell RNA sequencing datasets generated in this study are deposited in the Gene Expression Omnibus (GEO) with accession number GSE301633. All other data supporting the findings of this study are available from the corresponding authors upon reasonable request.

Research involving human participants, their data, or biological material

Policy information about studies with human participants or human data. See also policy information about sex, gender (identity/presentation), and sexual orientation and race, ethnicity and racism.

Reporting on sex and gender	N/A
Reporting on race, ethnicity, or other socially relevant groupings	N/A
Population characteristics	N/A
Recruitment	N/A
Ethics oversight	N/A

Note that full information on the approval of the study protocol must also be provided in the manuscript.

Field-specific reporting

Please select the one below that is the best fit for your research. If you are not sure, read the appropriate sections before making your selection.

☒ Life sciences ☐ Behavioural & social sciences ☐ Ecological, evolutionary & environmental sciences

For a reference copy of the document with all sections, see nature.com/documents/nr-reporting-summary-flat.pdf

Life sciences study design

All studies must disclose on these points even when the disclosure is negative.

Sample size	Sample sizes were based on prior experience with similar in vivo and imaging-based studies, balancing statistical sensitivity with ethical considerations of animal use. For key outcome measures (e.g. MRI, histology, scRNA-seq), biological replicates per group typically ranged from 3 to 8. This was sufficient to detect meaningful phenotypic differences in lymphatic growth, macrophage trafficking, and cardiac function, with statistical significance confirmed using appropriate tests (ANOVA, t-test, etc.).
Data exclusions	Data were excluded only when tissue quality was inadequate for analysis (e.g., poor sectioning, autofluorescence interference, failed immunostaining, or imaging artefacts). All exclusion decisions were made before analysis and without reference to group allocation or outcome. No animals were excluded based on phenotype or experimental outcome.
Replication	All key experiments were replicated in independent biological samples. For example, lymphatic growth was assessed across multiple litters at defined postnatal timepoints; adoptive transfer and imaging studies were repeated in at least two independent cohorts; and MRI and scRNA-seq analyses were replicated across multiple animals per genotype. All results presented are representative of findings reproduced in at least two independent experiments
Randomization	Animals were allocated to experimental groups based on genotype. Given the constraints of timed matings, litter availability, and the requirement for specific genotypes, formal randomisation was not applicable.
Blinding	Investigators were not blinded to group allocation during surgery or animal handling due to the technical nature of procedures (e.g., MI surgery at P1/P7). However, all image acquisition (e.g., confocal, light-sheet, MRI) and subsequent quantitative analyses (e.g., vessel metrics, immune cell counts, MRI readouts) were conducted in a blinded fashion.

Reporting for specific materials, systems and methods

We require information from authors about some types of materials, experimental systems and methods used in many studies. Here, indicate whether each material, system or method listed is relevant to your study. If you are not sure if a list item applies to your research, read the appropriate section before selecting a response.

Materials & experimental systems

n/a	Involved in the study	
☐	☒	Antibodies
☒	☐	Eukaryotic cell lines
☒	☐	Palaeontology and archaeology
☐	☒	Animals and other organisms
☒	☐	Clinical data
☒	☐	Dual use research of concern
☒	☐	Plants

Methods

n/a	Involved in the study	
☒	☐	ChIP-seq
☒	☐	Flow cytometry
☒	☐	MRI-based neuroimaging

Antibodies

Antibodies used

VEGFR3 R&D systems #AF743 Lymphatic endothelium 1:50 Goat (https://www.rndsystems.com/products/mouse-vegfr3-flt-4-antibody_af743)
LYVE-1 Angiobio #11-034 Lymphatic endothelium, tissue-resident macrophages, and endocardium 1:400 Rabbit (https://insightbio.com/productinfo/11-034/AngioBio)

PODOPLANIN Fitzgerald #10R-P155A Lymphatic endothelium and epicardium 1:200 Hamster (https://www.citeab.com/antibodies/10401-10r-p155a-podoplanin-antibody)

PHOSPHO-HISTONE H3 (PH3) Abcam #AB1791 Proliferation marker 1:200 Rabbit (https://www.abcam.com/en-us/products/primary-antibodies/histone-h3-antibody-nuclear-marker-and-chip-grade-ab1791?srsltid=AfmBOop1j-ysyQcC-ZEJQ7HC0Qb_RIgVQb48NywTWQwzvWOOa_xMba4z)

VE-CADHERIN R&D systems #AF1002 Adherens junctions 1:400 Goat (https://www.rndsystems.com/products/mouse-ve-cadherin-antibody_af1002)

PECAM1 BD Pharmingen #553370 Endothelium 1:200 Rat (https://www.bdbiosciences.com/en-us/products/reagents/flow-cytometry-reagents/research-reagents/single-color-antibodies-ruo/purified-rat-anti-mouse-cd31.553370?tab=product_details)

CD68 Bio-Rad #MCA1957 Macrophages 1:400 Rat (https://www.bio-rad-antibodies.com/monoclonal/mouse-cd68-antibody-fa-11-mca1957.html?f=purified)

IBA1 Abcam #ab5076 Macrophages 1:200 Goat (https://www.abcam.com/en-us/products/primary-antibodies/iba1-antibody-ab5076?srsltid=AfmBOookpLspaFFA61e3172IuX2dsuXfdWy4WGIWUA8ajnwguMasSgZv)

REELIN RndSystems #AF3820 Lymphatics 1:200 Goat (https://www.rndsystems.com/products/mouse-reelin-antibody_af3820)

ITGB1 Proteintech #12594-1-AP Endothelium, Macrophages 1:200 Rabbit (ITGB1 Proteintech #12594-1-AP)

CC3 Invitrogen #PA5-114687 Apoptotic Cells 1:200 Rabbit (https://www.thermofisher.com/antibody/product/Caspase-3-Cleaved-Asp175-Antibody-Polyclonal/PA5-114687)

CD44 eBioscience #14-0441-82 Leukocytes, Endothelium 1:200 Rat (https://www.thermofisher.com/antibody/product/CD44-Antibody-clone-IM7-Monoclonal/14-0441-82)

Hyaluronic Acid Binding Protein (HABP) Amsbio #AMS.HKD-BC41 Hyaluronic acid 1:300 Biotin conj. (https://www.amsbio.com/hyaluronan-binding-protein-habp-biotin-conj-ams-hkd-bc41)

AlexaFluor goat α-rabbit 405 Invitrogen 1 in 500
AlexaFluor donkey α-rabbit 405 Abcam 1 in 500
AlexaFluor donkey α-rat 488 Invitrogen 1 in 500
AlexaFluor donkey α-goat 488 Invitrogen 1 in 500
AlexaFluor donkey α-goat 555 Abcam 1 in 500
AlexaFluor goat α-rat 594 Invitrogen 1 in 500
AlexaFluor donkey α-goat 647 Invitrogen 1 in 500
AlexaFluor goat α-hamster 647 Invitrogen 1 in 500
AlexaFluor donkey α-rabbit 647 Invitrogen 1 in 500
Streptavidin, Alexa Fluor 488 Conjugate Invitrogen 1 in 200

Validation

All primary antibodies used in this study were commercially sourced and validated by the manufacturers for immunofluorescence in mouse tissue. Validation information, including species reactivity and application suitability, is available on the respective supplier websites and includes data from knockout controls or known positive/negative tissue staining (websites listed above). Where relevant, antibody specificity was supported by staining patterns consistent with published literature or prior in-lab use (e.g., LYVE-1 for lymphatic endothelium and tissue-resident macrophages; CD68 and IBA1 for macrophages; VE-Cadherin for endothelial

junctions). For all primary antibodies, secondary-only controls were used during protocol optimisation to exclude non-specific signal from secondary antibodies. No unexpected off-target staining was observed under the conditions used.

Animals and other research organisms

Policy information about studies involving animals; ARRIVE guidelines recommended for reporting animal research, and Sex and Gender in Research

Laboratory animals	For experiments where wild type mice were required C57BL/6 or CD1 (Charles River Laboratories) strains were used when indicated . Neonatal mice between P1 and P30 were used for experiments.
Wild animals	N/A
Reporting on sex	Both male and female neonatal mice were used in all experiments. Sex was not determined at the time of surgery or tissue collection due to the technical limitations of reliably sexing mice at early postnatal stages (P1–P7). Given the focus on early developmental windows and the absence of known sex-specific differences in lymphatic or macrophage responses during this period, data were pooled across sexes. Sex-based analysis was therefore not performed.
Field-collected samples	N/A
Ethics oversight	All animal experiments were carried out according to UK Home Office project licences PPL PC013B246 and PDDE89C84 and were compliant with the UK Animals (Scientific Procedures) Act 1986.

Note that full information on the approval of the study protocol must also be provided in the manuscript.

Plants

Seed stocks	N/A
Novel plant genotypes	N/A
Authentication	N/A
